# ADP-Ribosylargininyl reaction of cholix toxin is mediated through diffusible intermediates

**DOI:** 10.1186/s12858-014-0026-1

**Published:** 2014-12-11

**Authors:** Vicky M-H Sung, Chia-Lun Tsai

**Affiliations:** Center for Computational and Integrative Biology, Massachusetts General Hospital, Boston, MA 02114 USA; Department of Genetics, Harvard Medical School, Harvard University, Boston, MA 02115 USA

**Keywords:** Cholix toxin, ADP-ribosylation, Auto-ADP-ribosylation, Arginine

## Abstract

**Background:**

Cholix toxin is an ADP-ribosyltransferase found in non-O1/non-O139 strains of *Vibrio cholera*. The catalytic fragment of cholix toxin was characterized as a diphthamide dependent ADP-ribosyltransferase.

**Results:**

Our studies on the enzymatic activity of cholix toxin catalytic fragment show that the transfer of ADP-ribose to toxin takes place by a predominantly intramolecular mechanism and results in the preferential alkylation of arginine residues proximal to the NAD^+^ binding pocket. Multiple arginine residues, located near the catalytic site and at distal sites, can be the ADP-ribose acceptor in the auto-reaction. Kinetic studies of a model enzyme, M8, showed that a diffusible intermediate preferentially reacted with arginine residues in proximity to the NAD^+^ binding pocket. ADP-ribosylarginine activity of cholix toxin catalytic fragment could also modify exogenous substrates. Auto-ADP-ribosylation of cholix toxin appears to have negatively regulatory effect on ADP-ribosylation of exogenous substrate. However, at the presence of both endogenous and exogenous substrates, ADP-ribosylation of exogenous substrates occurred more efficiently than that of endogenous substrates.

**Conclusions:**

We discovered an ADP-ribosylargininyl activity of cholix toxin catalytic fragment from our studies in auto-ADP-ribosylation, which is mediated through diffusible intermediates. The lifetime of the hypothetical intermediate exceeds recorded and predicted lifetimes for the cognate oxocarbenium ion. Therefore, a diffusible strained form of NAD^+^ intermediate was proposed to react with arginine residues in a proximity dependent manner.

**Electronic supplementary material:**

The online version of this article (doi:10.1186/s12858-014-0026-1) contains supplementary material, which is available to authorized users.

## Background

ADP-ribosyltransferases (ADPRTs) constitute a broadly distributed family of prokaryotic and eukaryotic enzymes that catalyze the transfer of an ADP-ribose moiety from NAD^+^ to one or more substrates. The protein substrates of ADPRTs are heterogeneous and include eukaryotic elongation factor 2 (eEF2) [1,2], actin [3], vimentin [4], ezrin/radixin/moesin proteins [5], various heterotrimeric and small monomeric GTPases [6,7], the adapter proteins CRK-I and CRK-II [8], human α-defensin (HNP-1) and human β-defensin-1 (HBD1) [9,10], and cell surface molecules [11,12]. A family of ADPRTs ADP-ribosylate eEF2 on a post-translationally modified histidine residue called diphthamide [13]. Other ADPRTs transfer ADP-ribose to arginine, cysteine, glutamate, or asparagine residues [14,15]. Many ADPRTs also possess NAD^+^ glycohydrolase activity attributed to the attack by hydroxyl ion on the activated NAD^+^ transition state [16]. A number of ADPRTs exhibit auto-ADP-ribosylation activity, including diphtheria toxin [17,18], cholera toxin [19], *Clostridium limosum* C3 exoenzyme [20], *Pseudomonas aeruginosa* exoenzyme S [21], *Escherichia coli* heat-labile enterotoxin [22], *Neisseria meningitidis* ADP-ribosylargininyl transferase, *Nar*E [23], and several mammalian ADPRTs [24-26]. Auto-ADP-ribosylation of APDRTs has been shown to have functional consequences for the enzyme. For example, the auto-reaction of poly-ADP-ribose polymerase (PARP) has been reported to reduce its DNA-binding affinity, which in turn plays an important role in regulating the PARP response to DNA damage stimuli [27]. Similarly, auto-ADP-ribosylation of mammalian ADP-ribosyltransferase 5 (ART5) redirects the enzyme catalytic focus from NAD^+^ glycohydrolase activity to ADP-ribosyltransferase activity [28]. Auto-ADP-ribosylation has also been shown to inhibit the activity of the *C. limosum* C3 exoenzyme, *P. aeruginosa* exoenzyme S, and *Neisseria meningitides* ADP-ribosylargininyl transferase, *Nar*E [20,21,23].

Cholix toxin (*Chx*A) [15,29] and recently identified cholix toxin variants [30] were identified by genomic sequencing and homology comparisons in *Vibrio cholera* non-O1/non-O139 strains. Features of the primary sequences that were considered relevant to a phylogenetically conserved structural core supporting enzymatic function included an YXHG and an YXnY motif and a low but significant degree of homology to *P. aeruginosa* exotoxin A [15]. Many of the enzymatic attributes of cholix toxin have been established by the expression, catalytic characterization and determination at high resolution of a three dimensional crystal structure of the toxin and its catalytic subunit [2]. The results suggest that cholix toxin is a diphthamide-specific ADP-ribosyltransferase with a general domain organization and topology similar to that of exotoxin A. Members of the eEF2-specific ADPRT class are both phylogenetically and enzymatically distinct from other ADPRTs, and their common unique substrate, diphthamide, has been suggested to directly participate in the catalysis [31]. This class of ADPRTs has an elongated loop structure that is predicted or confirmed to significantly rearrange upon contact with eEF2 and that likely mediates the majority of interactions between enzyme and substrate [2,31,32]. Data from crystallographic and kinetic studies suggest the ADP-ribosylation of eEF2 is mediated through a random third order S_N_1 reaction, though the ribosyl-diphthamide bond adopts the α conformation consistent with the stereo inversion expected of an S_N_2 mechanism [33]. Here we characterized the process of the auto-ADP-ribosylation reaction of cholix toxin and evidenced that a metastable diffusible intermediate was generated upon the enzyme activation then diffused to react with arginine residues of the enzyme in a proximity dependent manner. We also showed that wild type cholix toxin catalytic fragment (abbr. as CTc) could ADP-ribosylate oligo argininyl peptides and eEF2 (H715R) mutant in which the post-translationally modified diphthamide at His715 was replaced by arginine. We propose that this mechanism can be used to engineer ADP-ribosyltransferases with alternative substrate specificity as long as there is an arginine residue or other ADP-ribose acceptors close to the catalytic site of the enzyme.

## Results and discussion

### An enzymatic pathway is involved in auto-ADP-ribosylation

**Figure 1:**
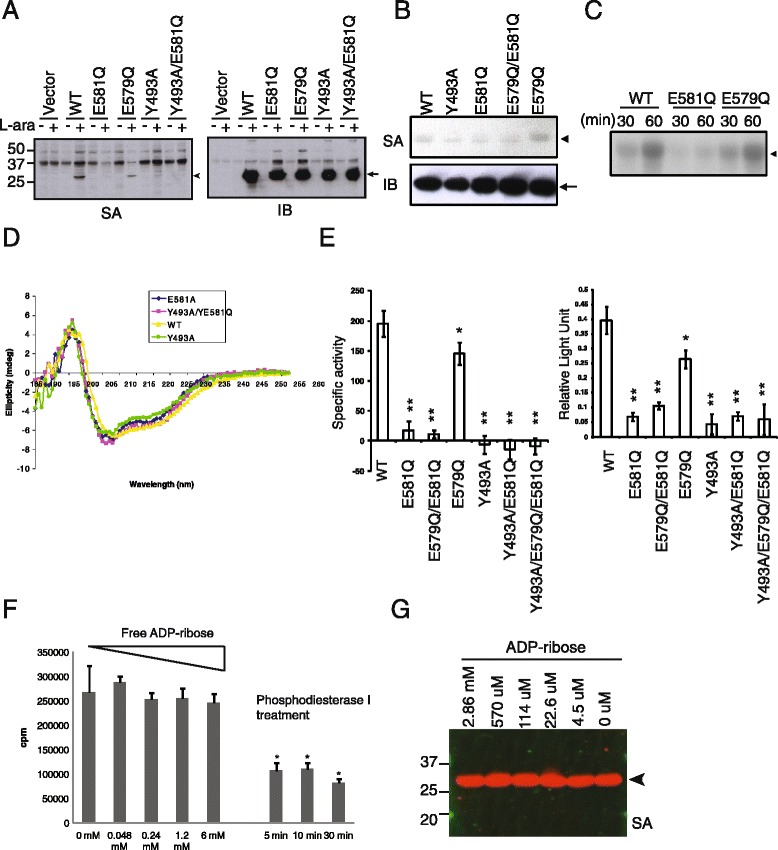
**An enzymatic pathway is involved in auto-ADP-ribosylation of CTc. (A)** Biotin signals are detected in the periplasmic fractions of *E.coli* lysate expressing wild type and mutant cholix toxin catalytic fragments (SA). The same blot was re-blotted with rabbit anti-CTc antibody to detect protein expression in each sample (IB). Similar results are obtained by incubating purified protein with either biotinyl-NAD^+^ (**B,** SA) or ^32^P-NAD^+^ **(C)**. The blots shown are representative of multiple independent experiments. **(D)** The structures of these mutants were analyzed by circular dichroism (CD) spectrometry. **(E)** NAD^+^ glycohydrolase activity (left panel) and auto-ADP-ribosylation activity (right panel) were quantified by 96-well plate based assays. Data are summarized from three independent experiments. Error bars show the standard deviation of the composite data. Asterisks indicate significant reduction of enzyme activity as compared to the wild type CTc with *, p-value < 0.004; **, p-value < 0.0001. **(F)** Various concentrations of free ADP-ribose were added to the auto-ADP-ribosylation reaction. As a control, the ^32^P-ADP-ribosylation signals on CTc were removed by phosphodiesterase I treatment. Asterisk indicates significant reduction of ^32^P-auto-ADP-ribosylation signal of the treated over the untreated samples with p-value < 0.01. Data shown is representative of three independent experiments. Error-bars show the standard error of mean (SEM). Statistic analysis was done by student *t*-test. **(G)** Excess amount of free ADP-ribose was added to the ADP-ribosylation buffer containing 3 μM purified wild type CTc and 50 μM biotinyl-NAD^+^ at 37°C for 1 hour. The biotinyl-ADP-ribosylation signal was detected by IRDye800CW-SA. In all panels, arrowheads indicate the auto-ADP-ribosylated CTc and arrows indicate the detection of CTc by anti-CTc antibody.

To characterize the enzymatic activity of cholix toxin we expressed the catalytic fragment under the control of the araBAD promoter as a translational fusion to a secB pathway-dependent signal peptide and assessed the ability of the periplasmic fraction to carry out ADP-ribosylation using biotinyl-NAD^+^ as a biotinyl-ADP-ribose donor. Initial experiments revealed the presence of an arabinose-inducible biotin-labeled band corresponding to the molecular weight of the catalytic fragment. Mutants Y493A, E581Q, and Y493A/E581Q (abbr. as YEDQ in the following) exhibited reduced biotinylation intensity compared to wild type and mutant E579Q, suggesting the catalytic fragment was capable of utilizing itself as a substrate (Figure 1A). The biotin labeled or ^32^P-labeled purified enzymes were also observed (Figure 1B and C), suggesting that host factors are not required. The possibility that the mutant forms had undergone structural changes resulting in a loss of substrate potential was minimized by the finding that the circular dichroism spectra of wild type and mutant proteins were substantially identical (Figure 1D). The auto-ADP-ribosylation and NAD^+^ glycohydrolase activities of the purified enzymes were also assessed under comparable conditions by a fluorescence-based assay (Figure 1E). The results indicate that the auto-ADP-ribosylation and NAD^+^ glycohydrolase activities of CTc are highly concordant. Mutations on any one of the conserved residues involved in catalysis resulted in loss of the auto-ADP-ribosylation activity (Figure 1A-C; Additional file 1A). These residues include E581, the catalytic residue; Y493 and Y504, two tyrosine residues binding to the aromatic group of NAD^+^; H460, providing the structural integrity of the catalytic site [15].

**Figure 2:**
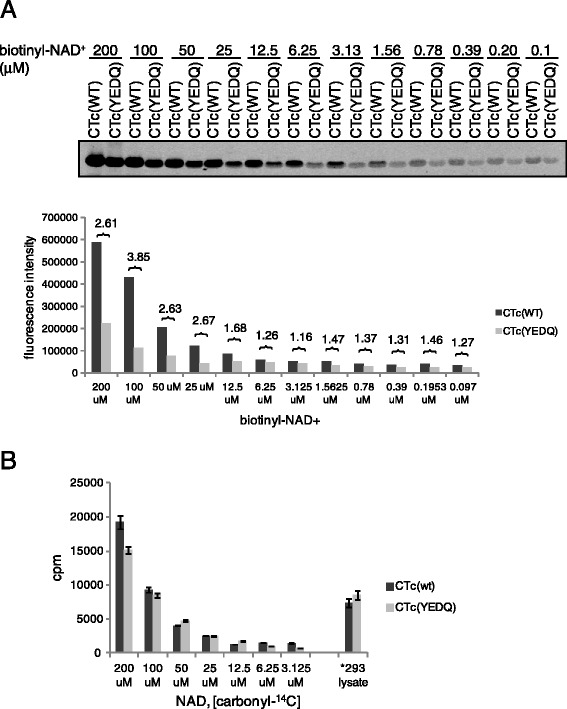
**Biotin signals on the self-modified wild type CTc is an ADP-ribosylation reaction. (A)** Purified wild type CTc or inactive mutant CTc(YEDQ), was incubated with various concentrations of biotinyl-NAD^+^. The folds of fluorescence intensity increase between CTc and CTc(YEDQ) were shown on the top of each pair of bar graph. **(B)** Purified CTc or CTc(YEDQ) was incubated with various concentrations of [carbonyl-^14^C]-NAD^+^ at 37°C for 1 hour. Asterisk indicates the control group in which 293 cell lysate was added to CTc or CTc(YEDQ) to measure ^14^C incorporation into the TCA precipitants. Data were summarized from two separated experiments.

Excess free ADP-ribose (up to 125-fold above assay concentration for ^32^P-NAD^+^ or 636-fold for biotinyl-NAD^+^) did not significantly inhibit either ^32^P- or biotinyl-ADP-ribosylation signals in the auto-reaction of CTc, suggesting that a non-enzymatic pathway resulting from the initial generation of ADP-ribose by NAD^+^ glycohydrolase activity, followed by non-enzymatic addition, does not play a major role in contributing to the observed auto-ADP-ribosylation reaction (Figure 1F and G). However, we also observed increased biotin substitution of the inactive mutant CTc(YEDQ) that had been incubated with increasing amounts of biotinyl-NAD^+^, which may be contaminated with some reactive NAD^+^ intermediates or ADP-ribose from the synthesis of biotinyl-NAD^+^ to cause the increased background biotin signals without catalytic activity (Figure 2A). Biotinyl-NAD^+^ bears the biotin substitution on the adenosine moiety of NAD^+^, whereas [carbonyl-^14^C]-NAD^+^ has ^14^C labeling on the nicotinamide moiety. When biotinyl-NAD^+^ was substituted by [carbonyl-^14^C]-NAD^+^, there was no significant difference in ^14^C incorporation between active and inactive enzymes even when as high as 100 to 200 μM of [carbonyl-^14^C]-NAD^+^ was used in the auto-reactions (Figure 2B). In contrast, active enzyme CTc showed ~4-fold higher biotin substitution than inactive mutant CTc(YEDQ) when 100 μM biotinyl-NAD^+^ was used. Overall, these data suggest that the increase in biotin substitution caused by the enzymatic activity of CTc is an ADP-ribosylation reaction.

### Auto-ADP-ribosylation favors intramolecular transfer

**Figure 3:**
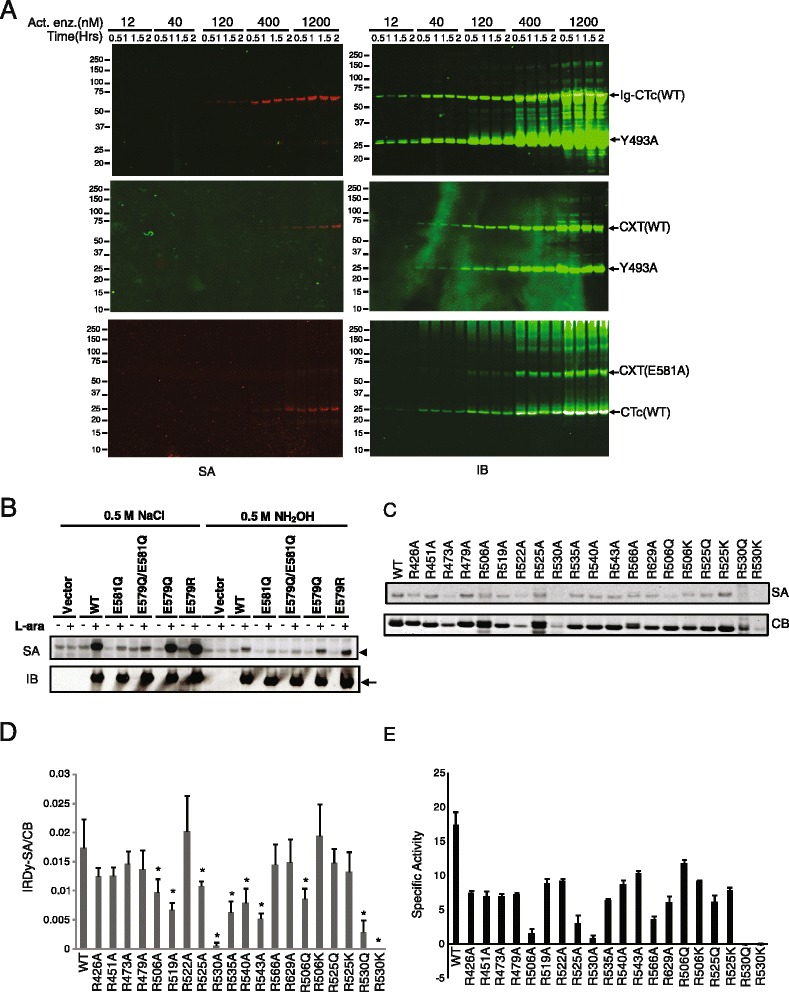
**Intramolecular transfer of ADP-ribose to multiple arginines in the auto-reaction. (A)** Mixing marked enzymes experiments. Active Ig-CTc was mixed with inactive mutant Y493A (top panel); active full-length cholix toxin, CXT (WT), was mixed with mutant Y493A (middle panel); inactive mutant CXT(E581A) was mixed with active CTc (bottom panel) in auto-ADP-ribosylation assays. Molar concentrations shown on the top of each blot are the concentrations of active enzyme. The left blots (SA) were detected by IRDye800CW-SA; the same blots were re-probed with polyclonal rabbit anti-CTc antibody shown on right (IB). **(B)** Neutral hydroxylamine assays. Auto-ADP-ribosylation reaction was carried out with periplasmic fractions of *E. coli* lysate expressing wild type or mutant CTc. The products were subjected to 0.5 M NH_2_OH (pH7.5) or 0.5 M NaCl treatment at 37°C for 2 hours. The blot shown is representative of multiple experiments. **(C-E)** Single amino acid substitution mutation studies. A set of representative data from the detection of biotin signals on wild type or different mutants was shown in **(C)**. The fluorescence intensity of SA (IRDy-SA) was normalized with the Coomassie Blue intensity (CB) to obtain the IRDy-SA/CB ratio as a semi-quantitative auto-ADP-ribosylation measurement of each enzyme. Data were summarized from four sets of experiments **(D)**. Asterisk indicates significant reduction of auto-ADP-ribosylation activity. NAD^+^ glycohydrolase activities of wild type and mutant enzymes are shown in **(E)**.

To understand if auto-ADP-ribosylation of cholix toxin involves intra- or inter-molecular modification, a catalytically active higher molecular weight form of CTc was prepared by fusion of the catalytic fragment to an immunoglobulin constant domain (abbr. as Ig-CTc), yielding an active enzyme with a molecular weight of 70 kDa. The resulting protein was mixed with mutant catalytic fragment in 1:5 molar ratio. The catalytically active form of the enzyme, Ig-CTc, was found to be preferentially substituted with biotinyl-ADP-ribose (Figure 3A). A reduced level of substitution with biotinyl-ADP-ribose was found for Y493A mutant protein that had been incubated at high concentration with Ig-CTc, which could be due to residual activity of the mutant enzyme or could reflect the occurrence of trans-ADP-ribosylation at high substrate concentration. Similarly, when purified recombinant full-length wild type cholix toxin (abbr. as CXT) was mixed with the catalytically inactive mutant, Y493A, or the catalytically inactive full-length cholix toxin E581A mutant was mixed with the wild type CTc, the auto-ADP-ribosylation of cholix toxin preferentially took place on the catalytically active form (Figure 3A). Together, these data support the view that auto-ADP-ribosylation of cholix toxin favors intramolecular transfer of the ADP-ribose moiety.

### Multiple arginines are the target residues

ADP-ribosyl adducts have different stabilities to neutral hydroxylamine: under conditions for which the half life of ADP-ribosylated glutamate is about 3–10 minutes, the half life of ADP-ribosylated arginine is about 120 minutes and the half lives of ADP-ribosylated cysteine and asparagine are immeasurably large [34]. As shown in the Figure 3B, the biotin substitution of the wild type and two active mutants, E579Q and E579R, showed no reduction following exposure to 0.5 M NaCl but fell by half after two hours of 0.5 M neutral hydroxylamine treatment at 37°C. Under these conditions there were little or no changes in the quantity or mobility of proteins as a result of either exposure. These data are consistent with the view that a significant fraction of biotinyl-ADP-ribose substitutions are the result of modification of arginine residues.

Since the ADP-ribosyl-glutamate linkage is labile in the neutral hydroxylamine treatment, we substituted two glutamate residues near the NAD^+^ binding pocket with lysine residues to create mutants E484K and/or E486K. None of these substitution mutations reduced the biotin signals on the auto-ADP-ribosylated enzymes (Additional file 1B). We therefore focus our study on the modification of arginine target residues.

To identify candidate residues for the auto-ADP-ribosylation, 14 arginine residues in the catalytic fragment were mutated to alanine residues. The extent of auto-ADP-ribosylation was significantly reduced after incubation of the R506A, R519A, R525A, R530A, R535A, R540A, or R543A mutant proteins with biotinyl-NAD^+^ substrate (Figure 3C and D), indicating these arginine residues may be the targets of auto-ADP-ribosylation. However, some mutant proteins also exhibited lower levels of NAD^+^ glycohydrolase activity than wild type catalytic fragment (Figure 3E) especially in the case of the R506A, R525A and R530A mutant proteins. Therefore, we replaced Arg506, Arg525, and Arg530 with glutamine or lysine, which has greater steric bulk than alanine (Figure 3C). The NAD^+^ glycohydrolase activity of R506Q, R506K, R525Q, or R525K was detectable at a level similar to that of other mutants and auto-ADP-ribosylation was also detected, suggesting other arginine residues than these can be ADP-ribosylated. All the attempts to replace Arg530 resulted in unstable enzymes with very poor activity and a low level of protein expression, indicating the enzyme is structurally sensitive to Arg530 modification. With the exception of Arg530 mutants, none of which were active, none of the single amino acid substitutions completely abolished auto-ADP-ribosylation, leads to the hypothesis that multiple arginine residues could serve as ADP-ribose acceptors.

### Identification of arginine target residues by 2-D electrophoresis and MS/MS analysis

**Figure 4:**
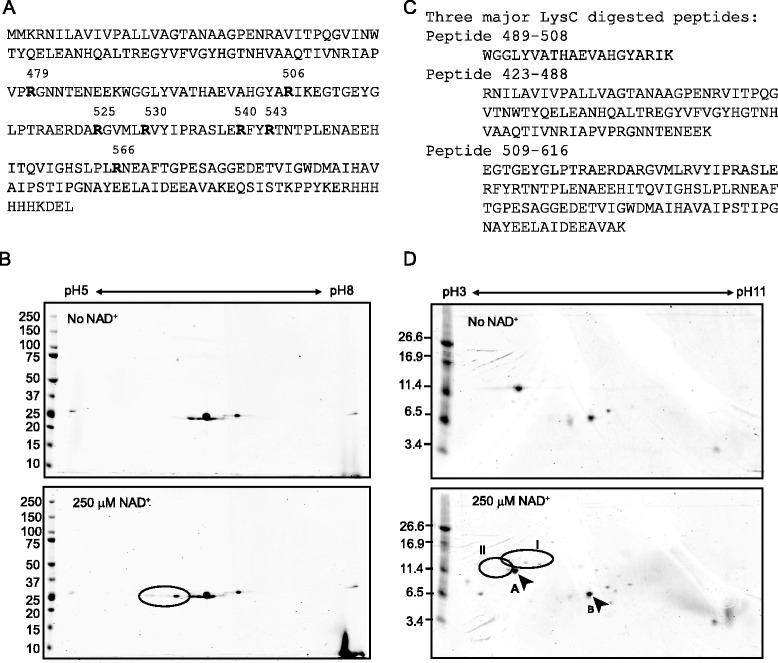
**Detection of multiple ADP-ribosylarginines by two-dimensional (2-D) electrophoresis.** The amino acids sequences of CTc fused with outer membrane signal peptide was shown in **(A)**. CTc was incubated with or without 250 μM NAD^+^ **(B)**. CTc incubated with or without NAD^+^ was further digested with LysC prior to peptide 2-D electrophoresis **(D)**. Amino acid sequences of three major predicted proteolytic products are shown in **(C)**. Circle I indicates the partially digested peptide 489–616. Circle II indicates spots shifted from the auto-ADP-ribosylated peptide 509–616. Spots A and B are peptides 509–616 and 423–488, respectively.

To support this hypothesis, we analyzed the auto-ADP-ribosylation of CTc using NAD^+^ as ADP-ribose donor by two-dimensional (2-D) electrophoresis. CTc has an isoelectric point (pI) of 5.5. One ADP-ribose addition to this fragment shifts its pI ~0.2 pH units. Following auto-reaction of the catalytic fragment, multiple spots shifted toward the acidic pH from the spot of CTc were detected by 2-D electrophoresis compared to the sample without incubation with NAD^+^ (Figure 4B, circled), indicating multiple residues being modified in the same protein. An additional spot found in the sample prior to incubation with NAD^+^ may result from auto-ADP-ribosylation *in vivo*, or from other post-translational modifications that occurred in the *E.coli* strain from which CTc was expressed. To map the location of the ADP-ribosylated arginines, the auto-ADP-ribosylated CTc was digested with LysC, predicted to cleave the CTc into three major peptides: peptide 423–488 (theoretical pI/MW: 5.85/7.4 kDa), peptide 489–508 (theoretical pI/MW: 8.51/2.2 kDa) and peptide 509–616 (theoretical pI/MW: 4.61/11.8 kDa). The LysC digested auto-ADP-ribosylated CTc was resolved in pH 3–10 matrices followed by 16.5% Tris-Tricine peptide gel analysis to separate the fragments. Two fragments (peptide 423–488 and peptide 509–616) were detected in gel spots B and A, respectively. Peptide 489–508 was too small to be observed in the peptide gel. However, a partial digest product spanning residues 489–616 was detected from which multiple shifted spots were detected (Figure 4D, circle I). At least three modified spots arising from the unmodified peptide 509–616 were detected in Coomassie Blue stained 2-D peptide gels (Figure 4D, circle II). These spots were missing from gels prepared from the CTc which was not incubated with NAD^+^. Peptide 509–616 contains several arginine residues (Arg525, Arg530, Arg540, Arg543 or Arg566) identified by reverse mutagenesis studies to be the targets for auto-ADP-ribosylation of CTc.

We also confirmed the ADP-ribosylarginine modifications by MS/MS analysis. The ADP-ribose moiety of ADP-ribosylated peptide is labile to the MS or MS/MS detection process. A two-step collision method was performed to remove the modification group (i.e., the ADP-R-carbodiimide moiety) with an in-source decay done in the tandem mass spectrometer [35]. The product from the first collision was then subject to the second collision to fragmentize the peptide chain with the small marker moiety to locate and determine the amino acid sequences. The combination of selection candidate peptides by the precursor ion scanning with ADP-R-carbodiimide as the marker ion and further fragmentation of the ion of the ADP-R-carbodiimide-derived peptides provided highly specific and reliable data applicable for MS/MS database searching for de novo peptide sequences. The ADP-ribosylargininyl peptide yields a product peptide in which arginine is replaced with ornithine (called Orn peptide) after first collision. From spot A (Figure 4D), we sequenced two Orn peptides, YGLPT/Orn/AERD and ARGVML/Orn/VYIPRASLE, indicating that the corresponding arginine positions Arg519 and Arg530 were ADP-ribosylated (Additional file 2). From the circled spots of Figure 4B and circles I and II of Figure 4D, four Orn peptide signals were detected to be Orn/FY/Orn/TNTPLE (for ADP-ribosylated Arg540 and Arg543), RFY/Orn/TNTPLE (for ADP-ribosylated Arg543), Orn/FYRTNTPLE (for ADP-ribosylated Arg540), and HITQVIGHSLPL/Orn/NEAFTGPE (for ADP-ribosylated Arg566). The Orn peptide containing Arg530 was unexpectedly detected in this study. The Arg530 position is sensitive to modification and the buried side-chain of Arg530 makes it unlikely to be ADP-ribosylated in its native conformation. We therefore reason the modification of Arg530 occurred artificially during the overnight iso-electrofocusing step when the enzyme was exposed to the 2-D sample buffer. The urea and detergent in the sample buffer could expose the side chain of Arg530 from its native position to react with the diffusible intermediate. Although we removed free NAD^+^ from the sample by gel filtration chromatography at the end of auto-ADP-ribosylation reaction prior to 2-D analysis, we may not have completely removed the enzyme bound fractions of NAD^+^. Arg530 is located close to the NAD^+^ binding pocket geometrically in the native 3-D structure of CTc.

### Identification of arginine target residues by direct and reverse mutagenesis

In our efforts to identify the auto-ADP-ribosylation target residues, a series of composite lysine to arginine replacement mutants were created as shown in Table 1, in which each successive mutant contained all the modifications of its parental template. Mutant M1 was constructed by introducing the R506K and R566K mutations into wild type catalytic fragment. M1 was used as a template to make mutant M2, which has additional R426K, R473K and R629K mutations. Arg530 was retained in all mutants because the protein integrity and catalytic activity are sensitive to its replacement. The mutants were purified and analyzed for their auto-ADP-ribosylation and NAD^+^ glycohydrolase activities (Table 1). Mutant M1 exhibited a reduction of biotin signal compared to wild type enzyme, implicating Arg506 and/or Arg566 as potential targets. Similarly, the degree of biotin substitution was decreased in M5-2, M4392, and M4421 compared to the parental M4 template, implicating Arg540 and Arg543 as potential targets. The reduction of biotinylation from M5-4 to M6 could be attributed to the R535K mutation and the reduction from M6 to M7 could be attributed to the R525K mutation. Introduction of R479K into the M7 template to make mutant M8 resulted in apparent abolition of biotinylation, suggesting Arg479 is an important target. However, when the R479K mutation was introduced into the context of mutant M4 to form M4781 mutant, significant biotinylation was detected, indicating Arg479 is not the sole ADP-ribose acceptor. The loss of substrate activity by mutant M8 coincided with a restoration of NAD^+^ glycohydrolase activity, suggesting the principal explanation for the diminished degree of biotinylation seen in this mutant is not extinction of catalytic activity (Table 1). Based on these data, Arg479, Arg506, Arg525, Arg535, Arg540, Arg543, Arg566 are potential target residues for auto-ADP-ribosylation.

**Table 1:** **Cholix toxin catalytic fragment variants contain arginine (R**) **to lysine (K**) **replacement mutations**

**R to K mutants**	**R to K replacement mutations on the wild type CTc**	^**ǂ**^ **Auto-ADP-ribosylation activity**	^**¶**^ **NAD** ^**+**^ **glycohydrolase activity**
M1	R506K,R566K	170	150.7 ± 1
M2	R426K,R473K,R506K,R566K,R629K	205	173 ± 3.4
M4	R426K,R473K,R506K,R519K,R522K,R566K,R629K	250	197.8 ± 4.6
M4392	R426K,R473K,R506K,R519K,R522K,R540K,R566K,R629K	113	105.4 ± 1
M4421	R426K,R473K,R506K,R519K,R522K,R543K,R566K,R629K	100	79.4 ± 5.9
M4781	R426K,R473K,R479K,R506K,R519K,R522K,R566K,R629K	130	126.6 ± 2
M5-2	R426K,R473K,R506K,R519K,R522K,R540K,R543K,R566K,R629K	100	7.8 ± 1.7
M5-4	R426K,R451K,R473K,R506K,R519K,R522K,R540K,R543K,R566K,R629K	155	8.2 ± 0.8
M6	R426K,R451K,R473K,R506K,R519K,R522K,R535K,R540K,R543K,R566K,R629K	90	9.6 ± 1.8
M6781	R426K,R451K,R473K,R479K,R506K,R519K,R522K,R535K,R540K,R543K,R566K,R629K	0	50 ± 1.5
M7	R426K,R451K,R473K,R506K,R519K,R522K,R525K,R535K,R540K,R543K,R566K,R629K	70	1 ± 3
M8	R426K,R451K,R473K,R479K,R506K,R519K,R522K,R525K,R535K,R540K,R543K,R566K,R629K	0	52.1 ± 1.7
WT	*-	215	206.3 ± 8.6

^ǂ^ Data show the mean fluorescence of intensity of biotin signals on the auto-ADP-ribosylated enzyme band detected by IRDye800-SA.

^¶^Data show the specific activity of each enzyme as described in Methods.

*Wild type cholix toxin catalytic fragment (WT) contains R426, R451, R473, R479, R506, R519, R522, R525, R530, R535, R540, R543, R566, R629.

**Figure 5:**
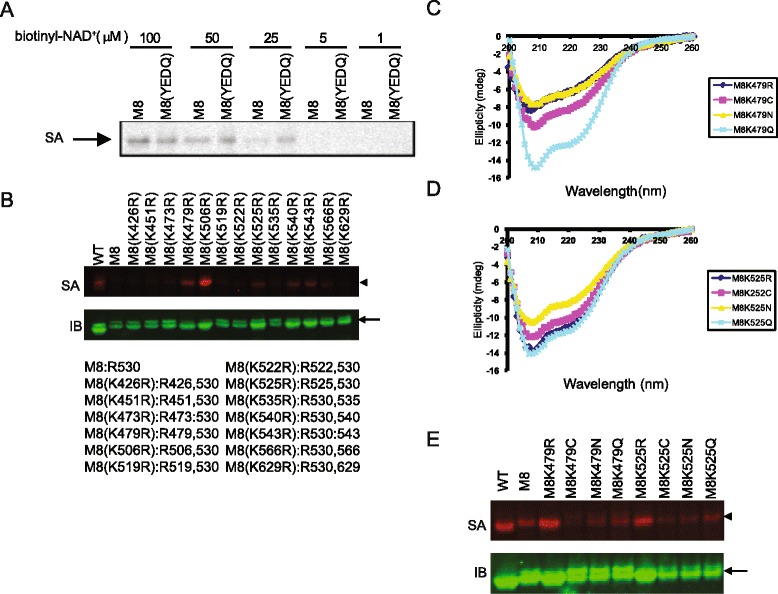
**Auto-ADP-ribosylation prefers arginines as target residues.** Mutant M8 contains 13 arginines (R) to lysines (K) replacement mutations except Arg530. **(A)** The same amount of M8 and its catalytically inactive form M8(YEDQ) were incubated with various concentrations of biotinyl-NAD^+^ in the auto-ADP-ribosylation reaction. The arrow indicates the biotinylated bands. **(B)** Mutant M8 was used as template for the K to R reverse mutation to determine which arginine(s) could be auto-ADP-ribosylated. The arginines present in the M8 and M8 derived mutant enzymes are indicated with their numerical positions in the full-length recombinant cholix toxin shown at the bottom of panel **B**. **(C-E)** Variation of acceptor residues were carried out by replacing Lys479 or Lys525 on the mutant M8 with arginine (R), glutamine (Q), asparagine (N), or cysteine (C). The circular dichroism of these M8 derived mutants were shown in **(C)** and **(D)**. Auto-ADP-ribosylation was performed with wild type or mutant cholix toxin catalytic fragments and detected by Western blotting shown in **(E)**. Arrowheads indicate biotinyl-auto-ADP-ribosylation of the catalytic fragments. Arrows indicate detection of the catalytic fragments by anti-CTc antibody for protein loading control.

Mutant M8 exhibited nearly undetectable auto-ADP-ribosylation and the background biotinyl signals of M8 were similar to those of the inactive mutant, M8(YEDQ) (Figure 5A). M8 mutant contains 13 arginines to lysines replacement mutations except Arg530. Arg530 has a buried (solvent non-exposed) side chain, which makes it less likely to be the target of auto-ADP-ribosylation in its native conformation. We therefore chose M8 as template to generate lysine (K) to arginine (R) reverse mutants. Among 13 K to R reverse mutants, M8(K479R), M8(K506R), M8(K525R), M8(K540R), M8(K543R) and M8(K566R) were all found to regain biotinylation signal intensity (Figure 5B), indicating multiple arginines can serve as ADP-ribose acceptors in the auto-ADP-ribosylation of M8 derived mutants. Together, we identified Arg506, Arg519, Arg525, Arg535, Arg540, Arg543 and Arg566 to be the target residues for auto-ADP-ribosylation.

### Diffusible intermediates

The locations of these arginine target residues are not restricted near the NAD^+^ binding pocket but also found at distal sites of the enzyme (PBD entry 2Q6M; [2]). In conjunction with the finding of intramolecular transfer of ADP-ribose to the active enzyme itself, we propose a mechanism by which a diffusible intermediate is created upon enzymatic activation and diffuses to react with arginine residues.

To further support this mechanism, we determined the auto-ADP-ribosylation rate of individual arginine residues using M8 K to R reverse mutants. Each M8 K to R reverse mutant contains Arg530 and one additional arginine residue. Using the method described in Kinetic Experiments, we compared the auto-ADP-ribosylation rates of six selected arginine target residues located at the proximal and distal sites of the NAD^+^ binding pocket (Table 2). M8(K506R) containing Arg506 target residue located at the end of the NAD^+^ binding loop had the highest auto-ADP-ribosylation rate, ~16-20-fold faster than the auto-ADP-ribosylation rates of M8(K519R) and M8(K525R). Under the assumption that these enzymes should have similar Km,_biotin-NAD+_ to make the correlation of the reaction rates with the distances of the acceptor residues in a simplified diffusion model, we normalized the Vmax of M6(K506R), M8(K519R), and M8(K525R) with their corresponding Km_, biotin-NAD+_, then the normalized auto-ADP-ribosylation rate of Arg506 is approximately 4 ~ 5-fold faster than those of Arg519 and Arg525, which are located on the substrate recognition loop near the NAD^+^ binding pocket. The auto-ADP-ribosylation rates of Arg540, Arg543 and Arg566, located at the distal or the opposite face of the NAD^+^ binding pocket, were low and highly variable among the inter- and intra-experimental repeats. Although the Km_,biotin-NAD+_ of M8(K543R) is 2-fold less than M8(K506R), the normalized auto-ADP-ribosylation rate (Vmax) of M8(K543R) is ~200-fold lower than that of M8(K506R), suggesting the electronic environment in the solution and the stability of the diffusible intermediate may affect the measured modification rates more pronouncedly in the auto-ADP-ribosylation of the three distal arginines. These data support that the auto-ADP-ribosylation is mediated through a diffusible intermediate that reacts with its target residues in a primarily proximity-controlled mechanism.

**Table 2:** **Kinetic analysis of auto-ADP-ribosylation of various arginines on M8 derived mutants**

	**Km**, _**biotin-NAD**_ **(μM)**	**Vmax (μM/min)**
**M8(K506R)**	31.7 ± 28.2	21767 ± 700.8
**M8(K519R)**	100 ± 121.8	1350 ± 997
**M8(K525R)**	79.3 ± 110	1005 ± 779.6
**M8(K540R)**	*n.d.	*n.d.
**M8(K543R)**	15.1 ± 25	56.6 ± 27.9
**M8(K566R)**	n.d.	n.d.

*n.d., cannot be determined. The values represent the mean ± standard error from 11 different experiments with two to three repeats in each experiment using GraphPad Prism 5.01.

To test what other amino acids the diffusible intermediate would react with, the Lys479 or Lys525 in M8 mutants were substituted with other potential ADP-ribose acceptors including cysteine, asparagine and glutamine. Most of these substitutions yielded significant secondary structure changes in the mutants. Mutant M8(K479R) showed a CD spectrum similar to M8(K479N) whereas M8(K525R) showed a CD spectrum similar to M8(K525Q), suggesting these mutants share similar structures (Figure 5C and D). However, neither M8(K479N) nor M8(K525Q) showed higher biotin substitution than M8(K479R) or M8(K525R) in the auto-ADP-ribosylation (Figure 5E). These data are consistent with previous reports that arginine is the preferred target residue for auto-ADP-ribosylation [20-22,26,28,36,37].

### Human ADPRH hydrolysis analysis

**Figure 6:**
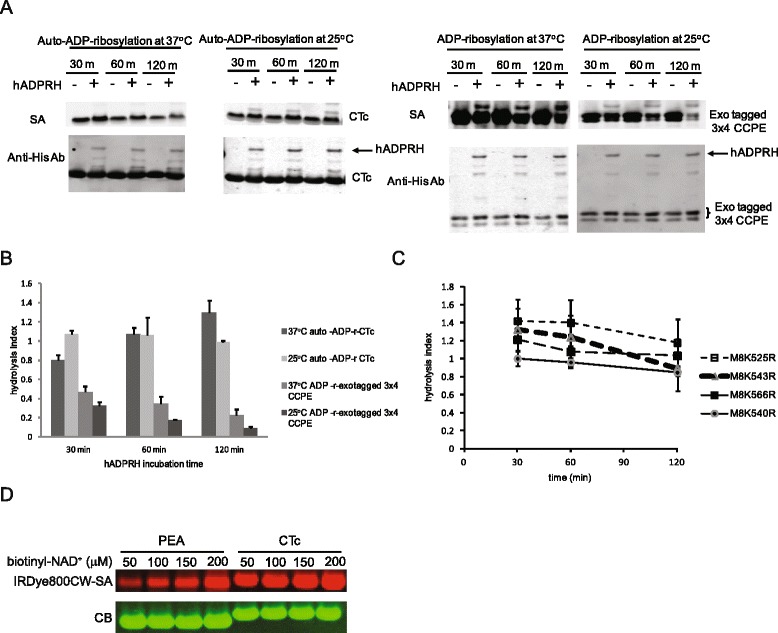
**Auto-ADP-ribosylation results a mixture of α** **- and** **β-anomeric ADP-ribosylargininyl adducts. (A)** Auto-ADP-ribosylated wild type CTc or M8 derived mutants or the ADP-ribosylated *exo*T substrate was incubated with purified hADPRH for various periods of time. The biotinyl-ADP-ribosylation remaining was detected by blotting with IRDye800CW-SA and quantified by Odyssey Infrared imaging system. The data shown in **(A)** are representative blots from multiple experiments. Five independent experiments were quantified and summarized in **(B)**. Data from 3 different experiments of hydrolysis of auto-ADP-ribosylated M8K525R, M8K543R and M8K566R were summarized in **(C)**. The error bar shows standard error of mean (SEM). Hydrolysis index is the ratio of normalized biotin signals remained on the hADPRH treated versus untreated substrates. **(D)** Auto-ADP-ribosylation of catalytic fragments of exotoxin A (PEA) and cholix toxin (CTc) was carried out with various concentrations of biotinyl-NAD^+^ in the ADP-ribosylation buffer containing 3 μM of purified enzymes shown in top panel. The bottom panel shows the Coomassie blue stained bands for protein loading control.

Human ADP-ribosylarginine hydrolase (hADPRH) has been shown to preferentially hydrolyze the α-anomer of ADP-ribosylarginine to release ADP-ribose from ADP-ribosylated protein [38]. *Pseudomonas aeruginosa* exotoxin T (*Exo* T) is an ADP-ribosyltransferase which specifically ADP-ribosylates arginine residues on target proteins in the presence of a host co-factor, FAS [8]. A minimal four amino acid peptide derived from Crk-I/II adaptor protein fused to the C-terminal domain of *Clostridium perfringens* enterotoxin (CCPE), named *exo*T substrate, was shown to be specifically ADP-ribosylated by ExoT/FAS complex (Tsai, manuscript in preparation). To understand the intermediate step of the auto-ADP-ribosylation of cholix toxin, we compared the activity of hADPRH on substrates formed by cholix toxin auto-activity or ExoT action on its substrate. Figure 6A and B show that hADPRH was able to effectively hydrolyze the biotinylated ADP-ribosylarginine from the ADP-ribosylated *exo*T substrate (> 80% reduction) whereas only 20-40% reduction of the auto-ADP-ribosylation of wild type CTc was observed after 30 minutes of hydrolysis reaction. Upon increasing hydrolysis incubation time, the biotin signals on the auto-ADP-ribosylated wild type CTc increased whereas the biotin-signals on the ADP-ribosylated *exo*T substrate decreased, suggesting the removal of ADP-ribose from the auto-ADP-ribosylated CTc may cause the enzyme to become more active. Wild type CTc has multiple arginines to serve as ADP-ribose acceptors. We speculated the increasing biotin substitution after hADPRH treatment could be attributed to more arginines being ADP-ribosylated at different sites of the enzyme. We therefore examined the hADPRH hydrolysis of the auto-ADP-ribosylated M8(K525R), M8(K543R) and M8(K566R), in which only two arginine residues are present in each mutant enzyme. The biotin signal of each auto-ADP-ribosylated M8 K to R mutant was reduced upon incubation with hADPRH though these mutants displayed different degrees of sensitivity to hADPRH hydrolysis (Figure 6C; Additional file 3). M8(K540R) and M8(K566R) exhibited a 10-20% of reduction in the auto-ADP-ribosylation signals after 120 minutes of incubation with hADPRH whereas M8(K525R) and M8(K543R) showed 40-50% reductions, possibly indicating steric factors may affect the accessibility of hADPRH to certain ADP-ribosylarginine residues.

Free ADP-ribose, generated by hADPRH hydrolysis activity, reacting with lysine to form Schiff-base adducts did not attribute to the increased resistance of auto-ADP-ribosylated CTc to hADPRH hydrolysis because no increases of resistance was observed in M8(K543R), M8(K540R), M8(K525R) nor in *exo*T substrate. ADP-ribosylation of *exo*T substrate at 37°C resulted in a small proportion of ADP-ribosylated *exo*T substrate (~20%) becoming more resistant to hADPRH hydrolysis than the *exo*T substrate that was ADP-ribosylated at 25°C. However, no difference was observed in the rates of hydrolysis between ADP-ribosylated *exo*T at 37°C and 25°C, suggesting no spontaneous anomerization occurred during the hADPRH hydrolysis reaction at 37°C. Thus, the possibility of spontaneous anomerization of α- to β-ribosyl linkage in ADP-ribosylarginine at 37°C, observed in choleragen [39], can not completely explain the degree of resistance to hADPRH hydrolysis seen in the auto-ADP-ribosylated CTc. Together, these data suggest that ADP-ribosylation carried by ExoT/FAS complex is an S_N_2-like reaction; whereas auto-ADP-ribosylation of CTc has mixed stereochemistry consistent with an S_N_1-like reaction mediated through diffusible intermediates.

### Auto-ADP-ribosylation model

To understand what this diffusible intermediate was, we first ruled out the possibility of the diffusible intermediate to be the ADP-ribose generated by the NAD^+^ glycohydrolase activity of the enzyme by free ADP-ribose competition assays shown in Figure 1F and G. Moreover, ADP-ribose has been shown to primarily interact with lysine, not arginine, residues at neutral pH. Decreases of biotin signals on a series of composed arginine to lysine substitution mutants was observed whereas lysine to arginine reverse mutants restored the biotin signals on the auto-ADP-ribosylated enzyme, indicating arginine is the primary target residue for the auto-ADP-ribosylation of CTc. However, we cannot exclude that lysine residue may contribute to some level of biotin signals appeared on the M8 mutant (Figure 5A).

The identification of the distal arginine residues, like Arg540, Arg543 and Arg566, being auto-ADP-ribosylated, suggests that this diffusible intermediate should be relatively stable in aqueous solution. The estimated half life of oxocarbenium ion, another well described intermediate, in aqueous solution is about 10 to 100 ps [40,41], which is too short to accommodate the time required for the intermediate to diffuse out of NAD^+^ binding pocket to react with the arginine target residues located at the distal sites before it reacts with water. Literature estimates for the NADH diffusion coefficient, 2.4×10^−6^ cm^2^ s^−1^ [42-44], predict that on the order of 4 to 400 ns would be required for the intact NAD^+^ molecule to diffuse 1 to 10 nm. Because rotational diffusion is typically orders of magnitude faster than translational diffusion and the predicted rotational relaxation time (rotational correlation time) of a protein with the approximate dimensions of CTc is about 15 ns [45], a combination of rotational and translational diffusions of both NAD^+^ and the enzyme would allow the distal sites on the opposite face of the enzyme to be reached within tens of nanoseconds. We thus hypothesize that the intermediate is a strained form of reactive NAD^+^. It is known that rate acceleration by enzymes could at least in part be due to introduction of strain into the part of the substrate which is undergoing reaction. The rate enhancement which is found upon introduction of strain into the substrate molecule by distortion can be very profound. This type of rate acceleration is thought to increase the energy of the ground state rather than to decrease the peak of the activation energy curve [46].

**Figure 7:**
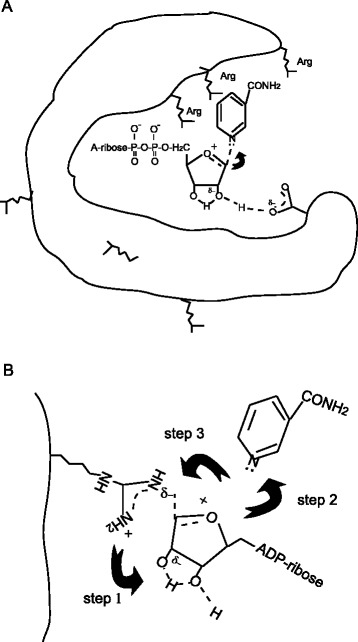
**Hypothetical model for auto-ADP-ribosylation. (A)** Formation of the diffusible strained NAD^+^ intermediate. At pH 7.5, the negatively charged carboxylate of the catalytic residue interacting with the 2′ hydroxyl group induced partial negative charges at the 2′hydroxyl position and formation of pseudo ribose diol to cause pre-protonation at C1′. This pre-protonated form of NAD^+^ is usually stabilized by the solvent molecules in its surroundings. We propose a mechanism in which this pre-protonated NAD^+^ intermediate gets released from the catalytic site and diffuses out of the NAD^+^ binding pocket prior to dissociation of nicotinamide when enzyme is busy in processing high concentration of NAD^+^. **(B)** Transfer of ADP-ribosyl moiety from strained NAD^+^ to arginine residue. This pre-protonated NAD^+^ intermediate approaches arginine residue through the positive charged side chain of arginine and interacts with the partially negatively charged pseudo ribose diol (step 1). Guanidinium group of arginine side chain contains three nitrogens. When one nitrogen is positively charged to interact with the ribose diol group, the induced partially negatively charged nitrogen (nucleophilic atom) interacts with the electrophilic C1 center of the N-ribose to cause dissociation of nicotinamide and formation of oxocarbenium ion which then immediately reacts with the partially negatively charged nitrogen on the guanidinium group of arginine to facilitate the transfer of ADP-ribose to the target arginine residue (step 2 and 3).

One obvious question is how this strained NAD^+^ intermediate was formed. We previously observed *Pseudomonas* exotoxin A has much lower auto-ADP-ribosylation activity than cholix toxin when similar levels of NAD^+^ substrate were used in the auto-ADP-ribosylation reactions. *Pseudomonas aeruginosa* exotoxin A, another member of the diphthamide-dependent ADP-ribosyltransferase family, shares 30% identity to cholix toxin and has similar numbers of arginines in the catalytic fragments (18 arginines in exotoxin A; 14 arginines in cholix toxin). If biotinyl-NAD^+^ by itself is capable of reacting with arginines, the same concentration of biotinyl-NAD^+^ used in the auto-reaction of exotoxin A and cholix toxin should yield similar level of biotin signals on both enzymes. Figure 6D shows that exotoxin A (Km_,ε-NAD_^+^ = 239.7 ± 89.79 μM) requires a much higher concentration of biotinyl-NAD^+^ to reach a similar level of auto-ADP-ribosylation signal as cholix toxin (Km,_ε-NAD_^+^ = 186.9 ± 87.79 μM). Km,_ε-NAD_^+^ of the enzyme by definition is the substrate concentration required to reach half of the maximal velocity of its NAD^+^ glycohydrolase activity. Previously, Oppenheimer proposed a model for the inductive stabilization of an oxocarbenium ion intermediate via an interaction between the 2′-hydroxyl and an active site carboxylate of an anionic residue at neutral pH, such as diphtheria toxin and exotoxin A, in an enzyme-mediated NAD^+^ hydrolysis mechanism [16]. Cholix toxin, like the other members of DT family, has glutamic acid (E) as its catalytic residue. Glutamic acid has a pKa of 4.8. At neutral pH, its carboxyl group is predominately negatively charged. Here we hypothesize that a strained NAD^+^ intermediate is pre-protonated at the catalytic site and released from the site before nicotinamide fully dissociated from the ribose while the enzyme is actively processing the substrate with its maximum speed (Figure 7A). The strained NAD^+^ intermediate then rotates to interact with arginine residues in proximity. This would provide an explanation for which exotoxin A required higher concentration of NAD^+^ substrate than cholix toxin did to reach similar level of auto-ADP-ribosylation when similar numbers of arginine target residues were available on both enzymes. Following the release of this pre-protonated NAD^+^ intermediate, the guanidinium group of arginine side chain (pKa ~ 12.43) approaching the partially negatively charged 2′-hydroxyl of the strained NAD^+^ intermediate leads to the complete dissociation of nicotinamide to form an oxocarbenium cation with an electrophilic C1 atom which then immediately reacts with the partially negatively charged nitrogen (nucleophilic atom) of the guanidinium group of the target arginine (Figure 7B) to finish the nucleophilic substitution reaction.

**Figure 8:**
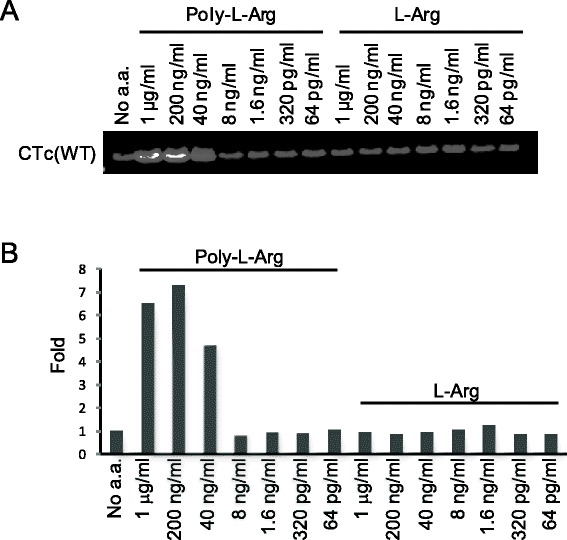
**Poly-L-arginines enhanced biotin signals on the auto-ADP-ribosylated wild type CTc. (A)** Various concentrations of free poly-L-arginines (Poly-L-Arg) or L-arginine (L-Arg) were added to the auto-ADP-ribosylation reaction of CTc and incubated at 37°C for 1 hr. **(B)** The biotinyl-auto-ADP-ribosylation signals were detected by Western blotting and presented as fold of change compared to the no amino acid added sample. The data shown is representative of three different experiments.

Figure 8 shows poly-L-arginines could enhance the auto-ADP-ribosylation of CTc up to 7–fold at the concentration of 200 ng/mL. Poly arginines are commonly found around the substrate binding domains of the ADP-ribosyltransferases known as arginine fingers. These arginines are located closely to the NAD^+^ binding pocket. The location of the poly arginines may alternatively provide an environment to facilitate the auto-ADP-ribosylation of the enzyme in the presence of NAD^+^ and absence of the exogenous substrate, like eEF2 for cholix toxin.

### ADP-ribosylation of endogenous versus exogenous substrates

**Figure 9:**
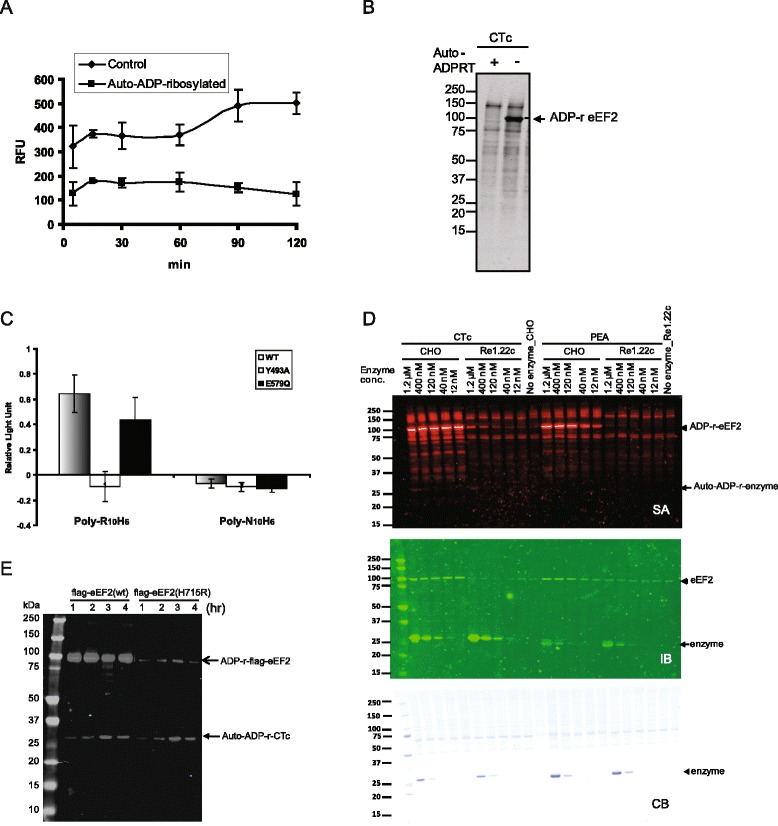
**ADP-ribosylation of endogenous versus exogenous substrates. (A-B)** Cholix toxin catalytic fragments were pre-incubated with or without 50 μM NAD^+^ at 37°C for 1 hour. Free NAD^+^ was removed by gel filtration chromatography. The recovered enzymes were quantified. Equal concentrations of auto-ADP-ribosylated CTc (pre-incubated with NAD^+^) or control (non-auto-ADP-ribosylated CTc, processed through auto-ADP-ribosylation reaction without NAD^+^) were used in the NAD^+^ glycohydrolase activity assays and ADP-ribosylation of eEF2 in 293 lysate. **(C)** His-tagged oligo-L-arginine or oligo-L-asparagine peptides were incubated with purified recombinant wild type CTc, catalytically defective mutant (Y493A) or catalytically active mutant (E579Q). The samples were analyzed by a 96-well plate based ADP-ribosylation assay. Data shown are composite from two experiments with triplicates within-plate replicates. **(D)**Various concentrations of catalytic fragments of cholix toxin or exotoxin A (PEA) was incubated with CHO or Re1.22c cell lysate at 37°C for 1 hr. The biotin signals on the ADP-ribosylated eEF2 and auto-ADP-ribosylated enzymes were detected by IRDye800CW-SA shown on the top panel. The same blot was stripped and re-probed with anti-CTc and anti-eEF2 antibodies shown in the middle panel. The bottom panel shows the Coomassie Blue stained gel for protein loading control. **(E)** To detect auto-ADP-ribosylation at the presence of exogenous substrates, excess amount of CTc (6 μM) was incubated with purified flag-tagged wild type eEF2 (0.2 μM) or flag-tagged eEF2 (H715R) mutant (0.2 μM) at the presence of 50 μM biotinyl-NAD^+^ for various periods of time.

Similar to several other ADP-ribosyltransferases [20,21,23], if we pre-incubated CTc with NAD^+^ to allow auto-ADP-ribosylation to occur prior to analysis, auto-ADP-ribosylation of the CTc suppressed its NAD^+^ glycohydrolase activity and ADP-ribosyltransferase activity to modify eEF2 (Figure 9A and B). Cholix toxin and exotoxin A are both characterized as diphthamide-dependent ADP-ribosyltransferases which modify eEF2 in nature. We also found that CTc could modify exogenous oligo-arginine peptides (Figure 9C). To understand how the enzyme ADP-ribosylates exogenous substrates in the presence of endogenous substrate, we mixed various amounts of purified catalytic fragments of cholix toxin or exotoxin A with CHO cell lysate, containing diphthamide-modified eEF2 or Re1.22c cell lysate, containing a mutated eEF2 without diphthamide modification, in the ADP-ribosylation reactions. The auto-ADP-ribosylation of the input enzyme was only found in highest concentration (1.2 μM) of CTc, whereas the ADP-ribosylation of wild type eEF2 has reached saturated signals with much lower concentrations of the enzymes. In this experimental setting, auto-ADP-ribosylation of exotoxin A was not detected even with 1.2 μM of the enzyme (Figure 9D). We further incubated purified flag-tagged eEF2(wt) or flag-tagged eEF2(H715R), with excess concentration of CTc to observe the kinetics of auto-ADP-ribosylation in the presence of exogenous substrates. The kinetics of ADP-ribosylation of flag-tagged eEF2 was much faster than that of auto-ADP-ribosylation of the enzyme or ADP-ribosylation of the flag-tagged eEF2(H715R) mutant, in which diphthamide was replaced with an arginine residue (Figure 9E). Diphthamide, which has imidazole-like structure on the modified histidine of eEF2, was shown to directly contact NAD^+^, and suggested to be involved in triggering the cleavage of NAD^+^ and interacting with the oxocarbenium intermediate during the nucleophilic substitution reaction [31]. We also observed low concentration of imidazole could enhance the biotinyl-ADP-ribosylation signals on the modified substrates (Additional file 4). These findings suggest that diphthamide can act as a catalyst to make ADP-ribosylation of eEF2 much more efficient than the auto-ADP-ribosylation reaction of cholix toxin. Moreover, at the presence of both endogenous and exogenous substrates, the binding of the exogenous substrate would segregate the arginine residues around the NAD^+^ binding pocket of the enzyme from interacting with the reactive strained NAD^+^ intermediate. Several arginine residues around the NAD^+^ binding pocket involved in substrate recognition and binding are also target residues of auto-ADP-ribosylation. Therefore, if the ADP-ribosylation of exogenous substrate results in lethal effect or triggers downstream of signal transduction pathways of the modified substrate, the negatively regulatory effect of the auto-ADP-ribosylation may be neglected and this is most likely to be true for most of bacteria toxins.

## Conclusions

Here we show that auto-ADP-ribosylation reaction of cholix toxin primarily involves the intramolecular transfer of ADP-ribosyl moiety to multiple arginine residues located around the NAD^+^ binding pocket as well as distal sites on the opposite face of the enzyme. This process is mediated through diffusible intermediates generated upon enzyme activation. Kinetic studies of M8 derived variants show the auto-ADP-ribosylation rates of the arginines at the proximal sites of the NAD^+^ binding pocket is significantly faster than those of the arginines located at the distal sites, supporting the diffusion mechanism. Owing to the extremely short half life of free oxocarbenium ion in aqueous solution, we hypothesize that the diffusible intermediate is a form of strained NAD^+^ which is pre-protonated at the catalytic site and released prior to the dissociation of nicotinamide to react with proximal arginine residues. This study elucidates the existence of a diffusible strained NAD^+^ intermediate that is different from the strained NAD^+^ alleviation model of the ADP-ribosylarginine reaction in the *Ita* toxin previously described [47,48]. We propose this mechanism can be applied to engineer artificial ADP-ribosyltransferases with alternative substrates by bringing substrates with arginine residues (or other nucleophilic target residues) close to the NAD^+^ binding pocket to react with diffusible intermediates generated by the ADP-ribosyltransferases.

## Methods

### Materials

6-biotin-17-NAD^+^ (abbr. as biotinyl-NAD^+^; Trevigen), 1,N^6^-ethenoadenosine5′-monophosphate disodium salt (abbr. as ethenoAMP; Sigma), nicotinamide 1,N^6^-ethenoadenine dinucleotide (abbr. ethenoNAD^+^; Sigma), β-nicotinamide adenine dinucleotide (MP Biomedicals, LLC), Adenosine 5′-diphosphoribose (abbr. ADP-ribose; Sigma), and Phosphodiesterase I from *Crotalus Adamanteus* venom (Sigma). Cholix toxin-specific antisera were raised by immunizing rabbits (abbr. as anti-CTc antibody, Affinity Bioreagent) with purified recombinant cholix toxin prepared in this study. All other reagents were from commercial sources unless specified.

### Cloning, expression and purification of recombinant proteins

**(i)** CTc: The 211-residue catalytic fragment of cholix toxin (GenBank accession no. AY876053) was assembled from chemically synthesized DNA oligonucleotides with a C-terminal 6xHis-tag (Genscript) and fused with outer membrane porin (*omp*F) leader sequences to target the protein expression to the periplasmic space of *E.coli*. The resulting fragment was inserted into a derivative of the pACYC184 vector in which the transcription of the inserted fragment was directed by the araBAD promoter under the control of the araC activator. The resulting prokaryotic expression plasmid was transformed into *E.coli* stain LC1062, which is a *recA*^*+*^*, endoA*^*−*^ version of *E.coli* strain MC1061. To induce recombinant protein expression one mL of overnight culture was inoculated into 100 mL of Terrific broth containing 12.5 μg/mL of chloramphenicol. The culture was grown to an OD_600_ of 0.5 at 37°C with vigorous shaking and cooled to 22°C prior to the addition of 0.5 mg/mL L-arabinose and further incubation at 22°C for 16 hours. The culture was harvested by centrifugation at 6000×g for 15 min and bacterial pellets were immediately frozen at −80°C. Ni-NTA spin columns were used to purify microgram amount of wild type and mutant catalytic fragment according to the manufacturer’s recommendation with slight modification. Briefly, spin columns were equilibrated with column buffer (50 mM NaH_2_PO4; 300 mM NaCl; 10 mM imidazole, pH 8.0). Bacterial pellets (1 g) were resuspended in one mL of Ni-NTA column buffer containing 1 mg/mL lysozyme and incubated on ice for 30 min. The lysate was homogenized and centrifuged at 14,000×g, 4°C for 30 min. The cleared supernatant was then loaded to a pre-equilibrated Ni-NTA spin column (Qiagen) and centrifuged at 700×g for 2 ~ 4 min at room temperature. The spin column then was washed twice with 600 μL of column buffer containing 20 mM imidazole. Finally, the protein was sequentially eluted with 200 μL of the column buffer containing 50 mM, 90 mM, and 250 mM imidazole. Five μL of each fraction was analyzed by SDS-PAGE followed by Coomassie Blue staining. The fractions containing purified protein were pooled and concentrated by Microcon YM-10 (Millipore) centrifugation. R530A, R530Q, R540K, and M8 derived mutants were purified by Ni-NTA spin column as described above, followed by Superdex200 (GE Healthcare) gel filtration chromatography purification in 10 mM HEPES buffer, pH 7.5 with a molecular weight about 26 kDa. **(ii)** CXT and Ig-CTc: CXT was codon optimized for *E.coli* expression and assembled from chemically synthesized DNA oligonucleotides with a C-terminal 6xHis-tag. The resulting DNA fragment was inserted into pET22(b) vector between *Nco I* and *Not I* sites. For the construction of Ig-CTc, sequences encoding a fusion protein were prepared by replacing the DNA sequence of domain I of cholix toxin with a DNA sequence encoding an anti-CD19 ScFv (HD37, peptide accession numbers CAA67618 and CAA67620) and inserting the combined sequence into pET22(b) vector between *Nco I* and *Not I* sites. An 18 amino acid linker (GGGGSGGGGSGGGGSGSS) joined the heavy chain and the light chain domains. The expression of CXT or Ig-CTc fusion protein in BL21(DE3) cells was induced with 0.2 mM isopropyl-β-D-thiogalactoside (IPTG) at 17°C overnight. A periplasmic fraction was prepared containing the fusion protein and further purified using Ni-NTA resin (Qiagen). Purified protein was dialyzed against PBS and stored at −80°C in small aliquots. **(iii)** hADPRH: The open reading frame of hADPRH was amplified from a cDNA clone of Open Biosystem collections by PCR with primers (5′-GGGATTCATATGGAGAAGTATGTGGCTGC-3′ and 5′-GGCTCGAGTAAGGGAAATTACAGTGTCTTC-3′) and cloned into the *Nde I* and *Xho I* sites of pET30a(+) vector. The plasmid was transformed into BL21(DE3) strain of *E.coli* and induced with 0.3 mM IPTG at 20°C for 16 hours. One gram of bacterial pellet was suspended in 4 mL lysis buffer (50 mM Tris, pH8.0; 150 mM NaCl; 3 mM β-mercaptoethanol (β-ME); 10 mM MgCl2; 0.1% (v/v) Triton X-100). After lysis by sonication, the cell debris was removed by centrifugation at 20,000×g at 4°C for 30 min. The clear supernatant was passed through Ni-NTA column and washed with 10 column volume of washing buffer (50 mM Tris, pH 8.0; 300 mM NaCl; 3 mM β-ME; 5 mM MgCl_2_), the protein was eluted in a buffer containing 50 mM Tris (pH8.0), 300 mM NaCl, 3 mM β-ME, 5 mM MgCl_2_ and 500 μM imidazole. The eluate was concentrated to a volume of 0.5 mL and directly applied onto a Superdex 200 size-exclusion column (GE Healthcare). Human ADPRH eluted in a buffer containing 50 mM Tris (pH 8.0), 150 mM NaCl and 2 mM DTT with a molecule weight about 40 kDa and was further dialyzed against 50% glycerol in 50 mM potassium phosphate buffer (pH7.5) for storage at −20°C. **(iv)** Expression and purification of *exoT* substrate and ExoT/FAS complex are to be described by Tsai (manuscript in preparation). Briefly, a tandem repeat of a minimal four amino acid peptide derived from Crk-I/II adaptor protein was fused to the C-terminal domain of *Clostridium perifringens* enterotoxin (CCPE) and the resultant fusion protein, named *exoT* substrate, was cloned into pET28 (a) vector with a 6xHis tag on the N-terminus. His-tagged ExoT or FAS was cloned into pET28 (a) and purified by Ni-NTA chromatography. The ExoT/FAS complex was co-purified by size-exclusion chromatography. **(v)** Flag-tagged eEF2 (wt) and eEF2(H715R) mutant. The flag-tagged wild type eEF2 was PCR-amplified from a cDNA clone of Open biosystem collections using primers: 5′-GGGAATTCGCCACCATGGACTACAAGGACGACGATGACAAGATGGTGAACTTCACGGTAGACC-3′ and 5′-GGGATCCGCGGCCGCTAGTGGTGGTGGTGGTGGTGCAATTTGTCCAGGAAGTTGTC-3′. The PCR product was inserted into a mammalian expression vector, pEAK15, on *EcoR* I and *Not* I sites. Mutant eEF2(H715R) was made by replacing diphthamide-modification target residue, His715, with arginine using primers: 5′-CCGACGCCATCCGGCGCGGAGGGGGC-3′ and 5′-GCCCCCTCCGCGCCGGATGGCGTCGG-3′, in a site-directed mutagenesis reaction. Ten μg of pEAK15-flag-eEF2(wt) or pEAK15-flag-eEF2(H715R) was transfected into a 10-cm plate of 85% confluent CHO cells with 40 μL of transfectin (Bio-rad). Forty-eight hours post-transfection, the cells were lysed in cell lysis buffer (50 mM Tris–HCl, pH 7.4; 100 mM KCl; 12.5 mM MgCl_2;_ 1 mM EDTA; 10% glycerol; 1 mM DTT; 0.1% NP40) containing EDTA-free protease inhibitor cocktails (Roche), and incubated on ice for 30 min prior to centrifugation at 14,000×g for 10 min. The supernatant was incubated with anti-flag M2 EZ-resin (Sigma) at 4°C overnight. The flag-tagged proteins were pulled down by centrifugation at 8,000×g for 30 sec and followed by washing the resin three times with the cell lysis buffer. The flag-tagged proteins were then eluted by 150 ng/mL of FLAG peptides (Sigma) in 20 mM Tris–HCl (pH 7.4).

### Mammalian cell culture

CHO and Re1.22c cells were cultured in Alpha-MEM (Invitrogen) containing 5% iron enriched calf serum (Hyclone). 293 cells were cultured in DMEM (Invitrogen) containing 10% iron enriched calf serum. CHO and Re1.22c cells were obtained from Dr. David Neville. All cell lines were cultured at 37°C, 5% CO_2_ incubator.

### Site-directed mutagenesis

All mutants used in this study were generated by using QuikChange Site-directed mutagenesis or QuikChange Multi site-directed mutagenesis kits (Agilent) according to the manufacturer’s instructions.

### Periplasmic fraction preparation

Fifty μL of overnight cultured *E. coli* transformants were inoculated into 5 mL of Terrific broth containing 12.5 μg/mL chloramphenicol. The cultures were grown to an OD_600_ of ~0.5 at 37°C and cooled to 22°C before addition of 0.5 mg/mL of L-arabinose. The cultures were then continuously cultured at 22°C for 16 hours before periplasm isolation. A 1 mL aliquot of each culture was pelleted by centrifugation at 14,000×g for 2 min. The pellet was suspended in 50 μL of periplasmic preparation buffer (200 mM HEPES-NaOH, pH7.5; 20% sucrose; 1 mM EDTA; 30,000 U/mL lysozyme) and incubated at room temperature for 5 min. Fifty μL of ice-cold water were added and mixed by gentle tapping followed by incubation on ice for 5 min. The periplasmic fraction was then separated from spheroplasts and bacterial debris by centrifugation at 10,000×g for 2 min and collection of the supernatant.

### Detection of auto-ADP-ribosylation of cholix toxin catalytic fragment

To detect the auto-ADP-ribosylated catalytic fragment in periplasmic fractions, 30 μg of periplasmic lysate was incubated with ADP-ribosylation buffer (1 mM EDTA; 1 mM DTT; 20 mM Tris–HCl, pH7.5) containing 50 μM biotinyl-NAD^+^ at 37°C for 1 hour. For detection of the ADP-ribosylation of purified protein, 2 μg of each purified enzyme was used. The reaction was terminated by adding SDS-PAGE sample buffer and boiled for 5 min. The samples were then resolved in 4-12% Criterion XT Bis-Tris gel with NuPAGE MES running buffer (Invitrogen). The gel contents were transferred to nitrocellulose and detected by blotting with either horseradish peroxidase (HRP)-conjugated SA (KPL) for chemiluminescence detection (PerkinElmer) or IRDye-800CW SA (LI-CORE Biosciences) for quantitation by Infrared imaging (Odyssey, LI-COR Biosciences).

For the radioactive detection of auto-ADP-ribosylation, 6 μM of purified enzyme was incubated with 50 μM ^32^P-NAD^+^ mix, which was prepared by dilution of [adenylate-^32^P]-NAD^+^ (800 Ci/mmol, PerkinElmer NEN) to a final specific activity of 3 Ci/mmol with unlabelled β-NAD^+^ (Sigma) to a final concentration of 100 μM NAD^+^ mix, at 37°C for 1 hour. The reaction was terminated by addition of 10 μg bovine serum albumin (BSA) and 10 μL of 100% tricholoroacetic acid (TCA) and incubated on ice for an hour, followed by centrifugation at 13,000×g for 10 min, washing the pellet once with 200 μL of ice-cold acetone and once with 200 μL of 95% ethanol, air-dry for 3 min prior to addition of protein sample buffer and boiled for 5 min. The samples were resolved by 10% Criterion XT Bis-Tris gel and the gel was heat-vacuum dried for 2 hours before exposure to Kodak storage phosphor screen. The image was scanned by Typhoon 9410 Variable Mode Imager and analyzed by ImageQuant software.

The auto-ADP-ribosylation reaction was also performed with various concentrations of [carobnyl-^14^C]-NAD^+^ in the ADP-ribosylation buffer with 6 μM of purified CTc or CTc(YEDQ) mutant. The reaction was terminated by addition of 10 μg BSA and 10 μL of 100% TCA and incubated on ice for an hour. Aliquots of 10 μL were spotted to Whatman 3 MM filter paper (0.75-inch square), washed twice with ice-cold 10% TCA and once in methanol (10 mL for each wash of a square filter paper; 2.5 min per wash). The level of radioactivity was counted in Beckman LS1801 multipurpose scintillation counter or PerkinElmer 2900TR Tri-Carb Liquid scintillation analyzer.

### Free ADP-ribose competition assays

**(i)** Radioactive detection: Various concentrations of free ADP-ribose were added in duplicates of 30 μL of auto-ADP-ribosylation reaction containing 7.6 μM purified CTc, 20 mM Tris–HCl (pH 7.5), 1 mM EDTA, 2 mM DTT, and 50 μM ^32^P-NAD^+^ mix. The auto-ADP-ribosylation reaction was carried out at 37°C for 90 min and the reaction was stopped by addition of 1 μL of 10 mg/mL BSA and 10 μL of 100% TCA, followed by incubation on ice for one hour. For the control experiment, the ^32^P-auto-ADP-ribosylated CTc was treated with 0.067 U/mL phosphodiesterase I at 37°C for up to 30 min, followed by addition of BSA and TCA on ice for 1 hour. Aliquots of 10 μL were spotted to Whatman 3MM filter paper, washed twice with ice-cold 10% TCA and once in methanol. The level of radioactivity was counted with 3 mL scintillation cocktail in Beckman LS1801 multipurpose scintillation counter. **(ii)** Western blotting detection: Various concentrations (4.5 μM to 2.86 mM) of free ADP-ribose were added to the auto-ADP-ribosylation reaction with 3 μM purified CTc, 50 μM biotinyl-NAD^+^. The biotinyl-auto-ADP-ribosylation signal was detected by Western blotting as described above.

### Immunoblot analysis

Wild type and mutant CTc or eEF2 in each transferred membrane were exposed to rabbit polyclonal anti-CTc antibody, rabbit polyclonal anti-eEF2 antibody (Abcam), or mouse anti-flag (M2) monoclonal antibody (Sigma) followed by different secondary antibodies. For hADPRH detection, rabbit polyclonal anti-hADPRH antibody (Absent) was used. For the detection of *exoT* substrate, rabbit polyclonal anti-his tag antibody (Santa Cruz) was used. Strong Re-blot buffer (Millipore) was used to stripe the membrane for re-blotting with different antibodies. For the chemiluminescence detection, HRP-conjugated goat anti-rabbit IgG (Promega) was used as secondary antibody; for the infrared imaging detection, IRDye680 conjugated goat anti-rabbit or IRDye680 conjugated anti-mouse antibody (LI-COR Biosciences) was used.

### Hydroxylamine sensitivity assay

To characterize ADP-ribosylated residues the auto-ADP-ribosylation reaction of wild type and mutant cholix toxin catalytic fragments were conducted at 37°C for 1 hour as described above. The reaction products were adjusted to 0.5 M NH_2_OH (pH7.5) or 0.5 M NaCl and incubated at 37°C for various periods of time. The reactions were terminated by addition of 4x sample buffer, boiled for 5 min and fractionated and blotted as described above.

### Plate-based auto-ADP-ribosylation assay

Wild type or mutant catalytic fragment (1.5 μg) was incubated with 50 μM biotinyl-NAD^+^, 10 mM HEPES-NaOH (pH7.5) and 2 mM MgCl_2_ at 37°C for 1 hour. The mixture was then added to a 96-well Ni-NTA plate and incubated for 2 hours on ice. The plate was washed with 1 M (NH_4_)_2_SO_4_ in PBS for 4 times, followed by washing with PBST (1% Tween 20 in PBS) twice. HRP-conjugated SA was then added to the plate and incubated for 30 min at room temperature. The plate was washed 8 times with PBST and developed by addition of 50 μL 1.25 mM tetramethylbenzidine (TMB) solution. The reaction was stopped by addition of 50 μL of 250 mM HCl and the absorbance measured at 450 nm.

### Detection of ADP-ribosylation of exogenous substrates

**(i)** ADP-ribosylation of artificial peptide substrates: The peptides R10H6 and N10H6 were chemically synthesized (Invitrogen). Purified enzyme (1.5 μg) was incubated with 1 mg/mL of R10H6 or N10H6 in a buffer containing 50 μM biotinyl-NAD^+^, 10 mM HEPES-NaOH (pH7.5) and 2 mM MgCl_2_ at 30°C for 30 min. The mixture was then added to a 96-well Ni-NTA HisPrime plate (5 PRIME Inc.) and incubated for two hours on ice. The plate was washed with 200 μL of 1 M (NH_4_)_2_SO_4_ in PBS for 4 times and 200 μL PBST twice. Fifty μL of 0.5 μg/mL HRP-conjugated SA was then added to the plate and incubated for 30 min at room temperature. The plate then was washed 8 times with 200 μL of PBST and developed by addition of 50 μL of TMB solution. The reaction was stopped by addition of 50 μL of 250 mM HCl and the absorbance measured at 450 nm. **(ii)** ADP-ribosylation of eEF2: CHO or Re1.22c cells were lysed in cell lysis buffer (50 mM Tris, (pH7.9); 100 mM KCl; 12.5 mM MgCl_2_; 1 mM EDTA; 10% glycerol; 1 mM DTT; 0.1% NP40) with protease inhibitors. For the ADP-ribosylation of endogenous eEF2 in cell lysate by the catalytic fragments of cholix toxin or exotoxin A, five μg of total cell lysate was added in a 25 μL ADP-ribosylation reaction with various concentrations of purified enzyme and the ADP-ribosylation assay buffer containing 50 μM biotinyl-NAD^+^. For the ADP-ribosylation of flag-tagged wild type or mutant eEF2, 0.5 μg of purified flag-tagged protein was incubated with 4 μg of purified CTc in a 25 μL ADP-ribosylation reaction containing 50 μM biotinyl-NAD^+^. The reaction was carried out at 37°C for various durations, and analyzed by Western blotting.

### Circular Dichroism (CD) spectrum analysis

CD spectra were recorded on a Circular Dichroism Spectrometer Model 202 (AVIV Instruments, Inc., Lakewood, NJ) in a quartz cell with an optical path of 1 mm at 25°C. Purified proteins were dialyzed against de-gassed 10 mM HEPES buffer (pH 7.5) and 5 μM of purified wild type and mutant CTc was used in the analysis. The samples were scanned from 185 or 200 nm to 300 nm with 1 nm increment. The signal was recorded from the average of 4 measurements for each wavelength with an averaging time of 5 seconds. Buffer control was scanned between each cell wash. The CD data of each sample scan was subtracted from the buffer only data collected before measurement of each protein sample.

### NAD^+^ glycohydrolase activity assays

A fluorometric assay based on ethenoNAD^+^ was used to quantify the NAD^+^ glycohydrolase activity of the wild type and mutant catalytic fragments [49]. In brief, in a 25 μL reaction, 6 μg of purified catalytic fragment was incubated with 0.4 mM ethenoNAD^+^ in a buffer containing 10 mM HEPES-NaOH (pH 7.5) and 2 mM MgCl_2_ at 37°C for 90 min. The reaction was stopped by addition of 100 μL of 12.2 mM EDTA in 10 mM HEPES-NaOH, pH7.5. The fluorescence was measured in a plate reader (Gemini EM, Molecular Devices) with excitation at 305 nm and emission at 410 nm and results were corrected for background fluorescence formed in the absence of enzyme to obtain the relative fluorescence units (RFU). EthenoAMP, used to generate the standard curve, was taken to have the same molar extinction and emission coefficients as ethenoADP-ribose. The specific activity was calculated in units/mg purified protein. One unit is defined as one nmol ADP-ribose formed in 90 min at 37°C.

### Kinetic experiments

Initial rate data for the auto-ADP-ribosylating specific arginines in M8 derived mutants were determined by incubation of 5 μM of purified enzymes with various concentrations of biotinyl-NAD^+^ from 5 to 100 μM in the ADP-ribosylation buffer at 37°C for 15 min. The reactions were immediately stopped and analyzed by Western blotting described above. The initial velocity (Vi,_15min_) of the auto-reaction in M8 or M8 derived mutants was calculated by the following equation: Vi_,15min_ = (FI,_15min_-FI,_0min_)/15 min. FI,_15min_ or FI,_0min_ is the fluorescence intensity of the auto-ADP-ribosylation signals semi-quantified by Odyssey Infrared Imaging system after 15 min incubation at 37°C, or at time 0, when various enzymes were boiled with protein sample buffer immediately after addition of ADP-ribosylation buffer containing various concentrations of biotinyl-NAD^+^. The rate of auto-ADP-ribosylation of Arg506, Arg519, Arg525, Arg540, Arg543 or Arg566 was determined by GraphPad Prism 5.01 using non-linear regression fitting of RVi,_15min_ in the Michaelis-Menten Model. RVi,_15min_ = (Vi,_15min_ of M8K506R, M8K519R, M8K525R, M8K540R, M8K543R or M8K566R mutant)- (Vi,_15min_ of M8 mutant).

### 2-D gel analysis of auto-ADP-ribosylated proteins or peptides

Eight μg of purified CTc was incubated at 37°C in a buffer consisting of 20 mM Tris, pH 7.5 and 250 μM NAD^+^. After 90 min, the reaction was stopped by removal of free unincorporated NAD^+^ using Illustra MicroSpin G-25 Columns (GE Healthcare). The sample was diluted in a rehydration/sample buffer (6 M urea, 1.5% CHAP, 37.75 mM DTT, 0.15% w/v Bio-Lyte 3/10 ampholytes, 5 mM Tris–HCl, pH 7.5 and trace amount of bromophenol blue) with 1:10 ratio. The diluted sample was evenly distributed to the IPG strip (pH 3–10 or pH 5–8) and overlaid with mineral oil to avoid evaporation. The rehydration of IPG strip was carried out in the PROTEIN IEF Focusing Tray in the PROTEIN IEF Cell under passive condition for 20 hrs before proceeding to the focusing stage. A three-step focusing method was used to program PROTEIN IEF Cell (step 1: voltage from 0 to 250 V, in 15 min with rapid ramp mode; step 2: voltage starts from 250 to 8000 V, in 2 hrs and 30 min with slow ramp mode; step 3: voltage is kept at 8000 V for 25,000 volt-hours with rapid mode) with the temperature set at 20°C and the current limit set at 50 μA/IPG strip. After the first dimensional isoelectric-focusing (IEF), the IPG strips (pH 3–10 or pH 5–8) were equilibrated in reducing buffer containing 6 M urea, 2% SDS, 0.375 M Tris–HCl (pH8.8), 20% glycerol and 2% DTT at room temperature for 15 min and followed by alkylation with 2.5% w/v iodoacetamide in 6 M urea, 2% SDS, 0.375 M Tris–HCl (pH8.8) and 20% glycerol at room temperature for an additional 15 min. The equilibrated IPG strip was placed horizontally on the Criterion XT 4-12% Bis-Tris (prep + 1 well Comb) gel and the proteins on the IPG strip were electrophoresed at 200 V for 45 min. For the peptide 2-D analysis, ten μg of auto-ADP-ribosylated CTc was incubated with 0.8 μg of LysC (Thermo Inc.) in 100 mM ammonium biocarbonate, pH 8.0 at 37°C for 6 hrs. The proteolytic products were separated by first-dimensional IEF using pH 3–10 IPG strips, followed by 16.5% Tris-Tricine peptide (prep + 2 well Comb) gel electrophoresis for second dimensional separation. The gels then were stained with Coomassie Brilliant Blue R-250.

### MS/MS analysis

Peptide from 2-D gel spots were analyzed by MS/MS analysis (Osago et al. manuscript in preparation). Briefly, each individual spot was destained, dried, and digested with V8 protease in 50 mM ammonium bicarbonate (pH 8.3) at 37°C overnight. Mass spectra of the digested peptides were acquired on a nanoLC-MALDI-TOF/TOF system (KYA Technologies DiNa nana-HPLC system-AB SCIEX TOF/TOF 5800) using 4000 Series Explorer (version 3.6). The data were analyzed with version 3.0 ProteinPilot^TM^ software.

### Human ADPRH hydrolysis assay

**(i)** Substrate preparation: Auto-ADP-ribosylation of CTc and M8 derived mutants were performed as the reaction described above at 37°C for an hour unless otherwise noted, followed by removal of free unincorporated biotinyl-NAD^+^ with NAP-5 columns (GE HealthCare). ADP-ribosylation of *exo*T substrate was performed by incubation of purified 2 μM *exo*T substrate with 50 nM ExoT, 200 nM FAS in PBS with 5 μM biotinyl-NAD^+^ at 25°C or 37°C for 1 hr. The reaction was terminated by removal of free unincorporated biotinyl-NAD^+^ with NAP-5 columns. **(ii)** Hydrolysis assay: The ADP-ribosylated substrates were incubated with 2.5 U of purified hADPRH in PBS buffer containing 10 mM MgCl_2_, and 5 mM DTT in a total volume of 25 μL for various time points at 37°C. Reactions were stopped by addition of protein sample buffer and boiling for 5 min. The sample was then subjected to SDS-PAGE and blot analysis as described above. One unit (U) of hADPRH defines the amount of purified hADPRH removal of greater than 70% of biotinyl-ADP-ribosylarginines from 0.5 μg of ADP-riobsylated *exo*T substrate in a 25 μL reaction at 37°C for an hour. **(iii)** Hydrolysis index: The spontaneous hydrolysis of ADP-ribosylarginines without hADPRH is observed in many *in vitro* ADP-ribosylation reactions. Continuous ongoing auto-ADP-ribosylation activity is also in some cases even after the removal of free biotinyl-NAD^+^ by gel filtration chromatography. Therefore, we define the hydrolysis index as the ratio of normalized fluorescent intensity (FI) of the hADPRH treated sample to the normalized FI of untreated sample. Normalized FI of each sample is the raw fluorescence intensity of IRDye800CW-SA divided by the raw fluorescence intensity of IRDye680. The hydrolysis index was then used to calculate the percentage of reduction of biotinyl-ADP-ribosylarginine signals after various periods of incubation time. The percentage of reduction then can be translated into the susceptibility of the ADP-ribosylargininyl adduct on each modified protein to hADPRH hydrolysis.

## Additional files

Additional file 1:
**Mutagenesis analysis for residues involved in auto-ADP-ribosylation of CTc.**
Additional file 2:
**MS/MS analysis spectra for de novo orn peptides sequencing.**
Additional file 3:
**Western blots for hADPRH hydrolysis of auto-ADP-ribosylated M8 K to R reverse mutants.**
Additional file 4:
**Imidazole effects on ADP-ribosylation.**

## Abbreviations

CTc: Cholix toxin catalytic fragment
CXT: Full-length cholix toxin
hADPRH: Human ADP-ribosylarginine hydrolase
Ig-CTc: CTc fused to an immunoglobulin constant domain
ethenoNAD: Nicotinamide 1,N^6^-ethenoadenine dinucleotide
ethenoAMP: 1,N^6^-ethenoadenosine 5′-monophosphate disodium salt
biotinyl-NAD^+^: 6-biotin-7-NAD^+^
ADP-ribose: Adenosine 5′-diphosphoribose
