# Supplementary material for: ADP-Ribosylargininyl reaction of cholix toxin is mediated through diffusible intermediates
Source: BMC Biochem. 2014 Dec 11;15:26. doi: 10.1186/s12858-014-0026-1 (PMC4265445; doi:10.1186/s12858-014-0026-1)
Supplement: Additional file 1: — Mutagenesis analysis for residues involved in auto-ADP-ribosylation of CTc. [file 12858_2014_26_MOESM1_ESM.pdf]

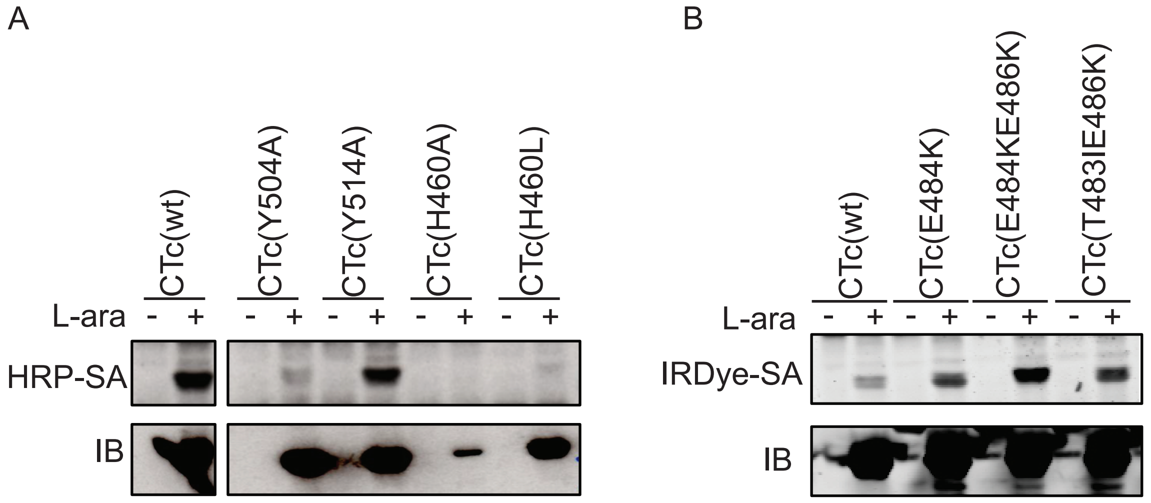

Additional file 1. Mutagenesis analysis. (**A**) Detection of auto-ADP-ribosylation signals on periplasmic lysates expressing wild type CTc, or mutant enzymes CTc(Y504A), CTc(Y514A), CTc(H460A) and CTc(H460L). Y504 is one of two tyrosine residues binding to NAD^+ ; Y514 is a tyrosine residue 10 amino acids downstream of the NAD^+ binding loop; H460 is a conserved residue providing the structural integrity of the catalytic site. (**B**) E484 and E486 are two glutamic acids residues located near the NAD^+ binding pockets. Mutations of both glutamic acids to lysine residues do not show any reduction of the biotin signals on the auto-ADP-ribosylated enzymes. L-ara indicates the periplasmic lysates with or without L-arabinose induction.
