# Supplementary material for: ADP-Ribosylargininyl reaction of cholix toxin is mediated through diffusible intermediates
Source: BMC Biochem. 2014 Dec 11;15:26. doi: 10.1186/s12858-014-0026-1 (PMC4265445; doi:10.1186/s12858-014-0026-1)
Supplement: Additional file 2: — MS/MS analysis spectra for de novo orn peptides sequencing. [file 12858_2014_26_MOESM2_ESM.pdf]

Additional file 2. MS/MS de novo peptide sequencing for Orn Peptide. (**A**) and (**B**) show the de novo peptide sequencing data of two Orn peptides YGLPTR(Orn)^{519}AERD and ARGVMLR(Orn)^{530}VYIPRASLE by MS/MS analysis. The table below panels **A** and **B** list the detected b and /or y ions matched to the residues of each peptide shown in the MS/MS spectra.

(A)

YGLPTR(Orn)^{519}AERD

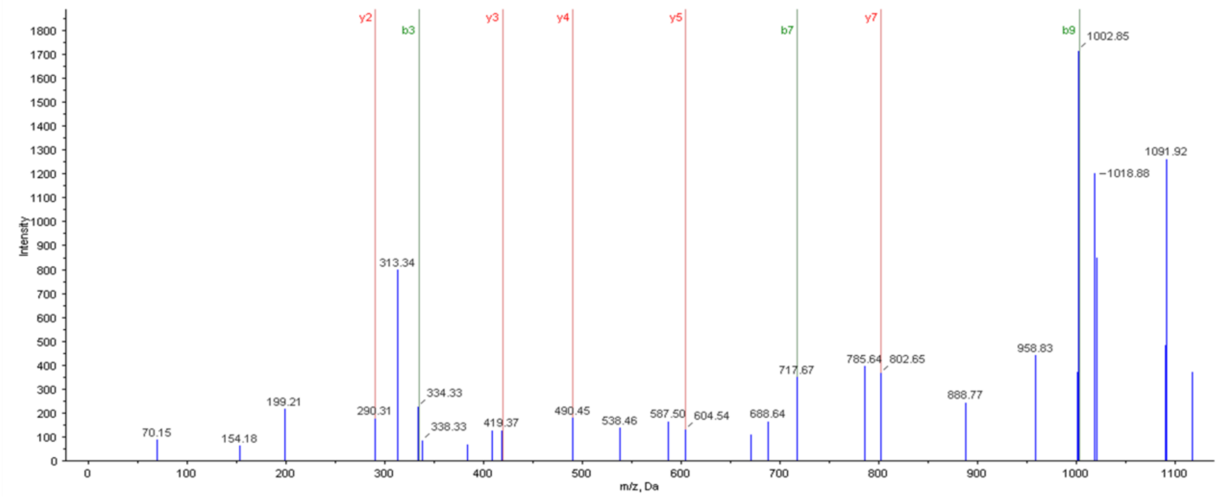

Residue	Y	G	L	P	T	R(Orn)	A	E	R	D
b			334.17				717.39	419.18	1002.53	
y				802.40		604.30	490.22		290.14	

(B)

ARGVMLR(Orn)^{530}VYIPRASLE

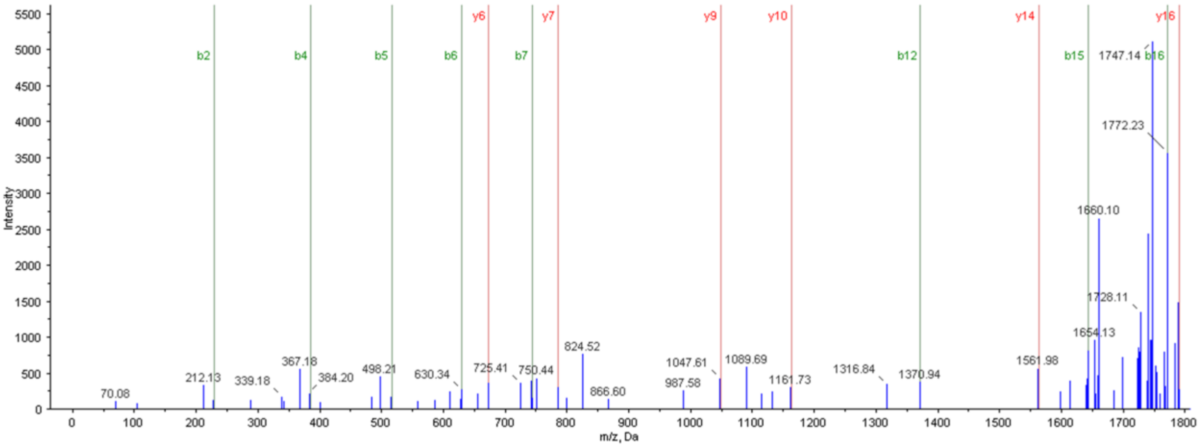

Residue	A	R	G	V	M	L	R(Orn)	V	Y	I	P	R	A	S	L	E
b		228.14		384.23	515.27	628.35	742.43					1370.80			1641.96	1771.00
y	1789.91		1561.87				1161.66	1047.58		785.45	672.36					
