# Supplementary material for: ADP-Ribosylargininyl reaction of cholix toxin is mediated through diffusible intermediates
Source: BMC Biochem. 2014 Dec 11;15:26. doi: 10.1186/s12858-014-0026-1 (PMC4265445; doi:10.1186/s12858-014-0026-1)
Supplement: Additional file 3: — Western blots for hADPRH hydrolysis of auto-ADP-ribosylated M8 K to R reverse mutants. [file 12858_2014_26_MOESM3_ESM.pdf]

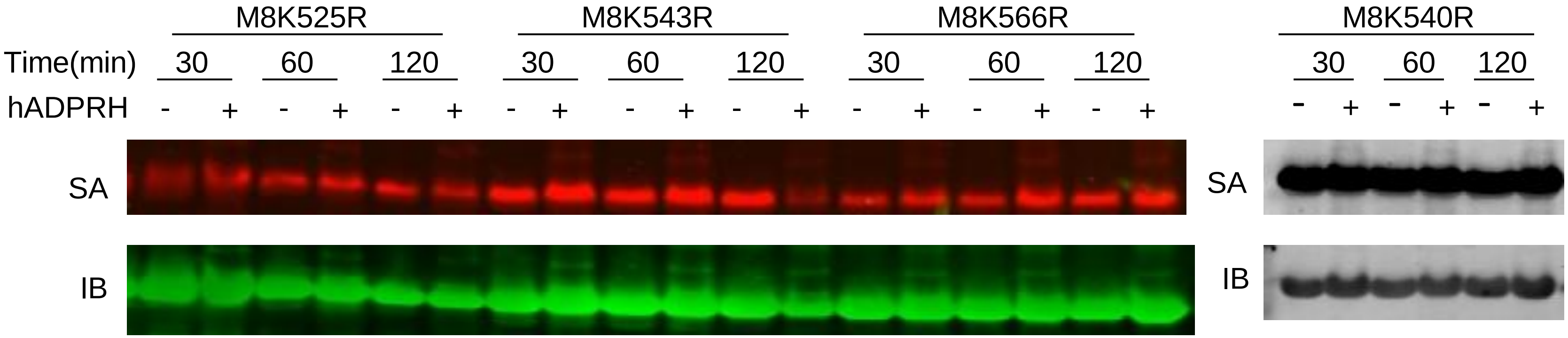

Additional file 3: One set of representative blots from hADPRH hydrolysis of auto-ADP-ribosylated M8K525R, M8K543R, M8K566R, and M8K540R shown in Figure 6C. SA indicates detection of the biotin signals remained on each band after incubation at 37°C with or without hADPRH. IB indicates the immuno-blotting detection of the amount of protein loaded in each band.
