# Supplementary material for: ADP-Ribosylargininyl reaction of cholix toxin is mediated through diffusible intermediates
Source: BMC Biochem. 2014 Dec 11;15:26. doi: 10.1186/s12858-014-0026-1 (PMC4265445; doi:10.1186/s12858-014-0026-1)
Supplement: Additional file 4: — Imidazole effects on ADP-ribosylation. [file 12858_2014_26_MOESM4_ESM.pdf]

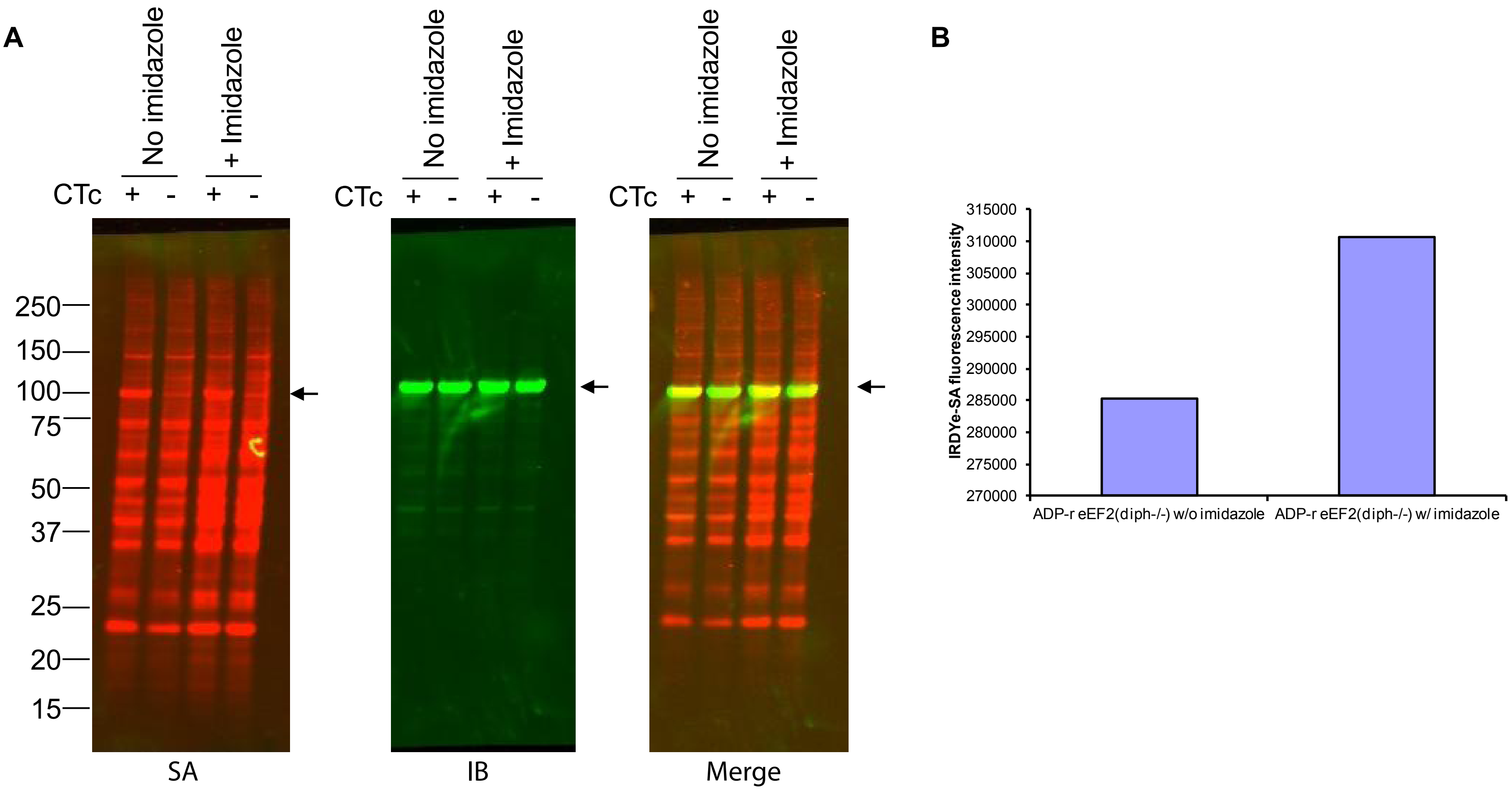

Additonal file 4. Imidazole enhanced ADP-ribosylation of diphthamide deficient eEF2 in PR72 cell lysate by CTc. (**A**) ADP-ribosylation of eEF2 from a DHP3 inactivated CHO cell line, PR72 cells (Gupta, P.K. et al, 2008. Cellular Microbiology), Left panel shows detection of biotin signals on the PR72 cell lysate incubated with CTc with or without imidazole. Middle panel shows detection of the eEF2 expression in the same blot with polyclonal anti-eEF2 antibody. Right panel shows the merged signal of left and right panels. Arrows indicate the eEF2 expressed in PR72 cell ine. (**B**) Quantification of the biotin signals of the ADP-ribosylated eEF2 detected in (**A**).
